# Enhancing vehicle routing problem through a qubit-efficient Symmetric Arc Reduction using Quantum Approximate Optimization Algorithm

**DOI:** 10.3389/frai.2026.1900887

**Published:** 2026-10-01

**Authors:** Jayaraja A, Senthil Kumaran U

**Affiliations:** 1 Department of Mathematics, School of Advanced Sciences, Vellore Institute of Technology, Vellore, Tamil Nadu, India; 2 School of Computer Science Engineering and Information Systems, Vellore Institute of Technology, Vellore, Tamil Nadu, India

**Keywords:** gate-based, qubit reduction, SAR-QAOA, Symmetric Arc Reduction, vehicle routing problem

## Abstract

**Introduction:**

Finding the most efficient routes for a fleet of delivery vehicles remains a critical and costly challenge for nearly any logistics business. This core idea is known as the Vehicle Routing Problem (VRP). The primary goal is to minimize operational costs, such as the total travel distance or the number of trucks deployed, while ensuring all the customers are served. Because VRP is NP-hard, finding a perfect solution is often impossible, forcing industries to rely on classical approximate algorithms. However, quantum variational algorithms offer a new paradigm by iteratively optimizing parameters to navigate the solution space, presenting a new and promising avenue for finding better and faster solutions.

**Methods:**

In this paper, we introduce Symmetric Arc Reduction in Quantum Approximate Optimization Algorithm (SAR-QAOA), which systematically eliminates redundant symmetric routing variables in the binary integer programming formulation prior to the Quadratic Unconstrained Binary Optimization (QUBO) formulation. The proposed SAR-QAOA encodes edge-based VRP while reducing the qubit requirement from *n*(*n*−1) to *n*(*n*−1)/2, thereby improving the resource efficiency of the QUBO formulation by prioritizing regions of the solution space that are more likely to contain high-quality solutions; SAR enhances both search accuracy and computational efficiency.

**Results:**

Experimental results on noiseless quantum simulators validate the feasibility of the proposed formulation and its integration with the QAOA framework, providing a promising foundation for future investigations on larger routing problems and evolving quantum hardware.

## 1 Introduction

In recent times, logistics has gained significant attention because of transportation expenses and longer travel distances, along with the potential to reduce costs in this sector and the urgent need to deliver products to market quickly. In logistics management, solving vehicle routing challenges is one of the most critical aspects; this is known as the Vehicle Routing Problem (VRP), in which vehicles start at the depot, visit one or more customers, and then return to the depot.

The Vehicle Routing Problem (VRP) is one of the most essential and widely examined challenges in combinatorial optimization, particularly within operations research and logistics management. The central challenge of the VRP is to find the optimal routes for a fleet of trucks to serve a set of customers. The objective is to minimize real-world costs such as fuel and driver time while adhering to constraints such as vehicle capacity and delivery windows. This problem generalizes the well-known Traveling Salesman Problem (TSP), which involves a single vehicle. Its formal study began in 1959, when Dantzig and Ramser first defined it as “The Truck Dispatching Problem” (Dantzig and Ramser, 1959). This state-of-the-art addressed the practical challenge of optimizing gasoline delivery routes from a bulk terminal to various service stations, effectively laying the foundation for what would become a critical field of study in transportation logistics. Building on this study, Clarke and Wright developed the most influential heuristic approach for VRP, widely known as the savings algorithm, in 1964 (Clarke and Wright, 1964). The formal designation “Vehicle Routing Problem” was adopted by Christofides (1976), marking a pivotal moment in standardizing the nomenclature and establishing VRP as a distinct research domain. The VRP has evolved from its initial conceptualization to encompass increasingly complex real-world scenarios and has become indispensable for optimizing logistics operations involving trucks, delivery vehicles, and other transportation systems. The most common variant is the Capacitated Vehicle Routing Problem (CVRP), which enforces vehicle capacity limitations and is the most researched variant. Furthermore, the Vehicle Routing Problem with Time Windows (VRPTW), where deliveries are required to happen within defined time frames; the Heterogeneous Vehicle Routing Problem (HVRP), featuring different fleet capacities; the Multi-Depot Vehicle Routing Problem (MDVRP); and the Two-echelon Vehicle Routing Problem (2E-VRP), which pertains to hierarchical distribution systems.

To address these variants, exact algorithms such as the Branch-and-Bound (Fischetti et al., 1994) technique can find optimal solutions using mathematical methods. However, as problem scale increases, they require high computational time due to exponential growth. Consequently, heuristic and metaheuristic methods have prevailed in practical applications, with traditional techniques like the Clarke-Wright savings algorithm, nearest neighbor heuristics, and two-phase construction algorithms delivering quick feasible solutions. Nevertheless, these algorithms are often sensitive to parameter tuning and prone to getting stuck in local optima. Despite their computational speed, they may fail to consistently converge to the global optimum within a limited feasible time span for highly complex landscapes.

Quantum computing offers a promising alternative for tackling large-scale intractable classical problems, such as VRP (Azfar et al., 2025). One of the most promising domains for this challenge is quantum optimization, a field that underpins numerous real-world applications such as logistics, resource allocation, machine learning, and network design (Slysz et al., 2026). Moreover, the notable advantage of quantum computing lies in its intrinsic parallelism, enabled by quantum mechanical principles such as superposition and entanglement, which substantially improves the efficiency of optimization computations and allows rapid determination of global minima and maxima (Dasari et al., 2020). Various methods have been proposed to address the vehicle routing problem using algorithms such as quantum annealing, the Quantum Approximate Optimization Algorithm, and the variational quantum eigensolver (Alsaiyari and Felemban, 2023). However, variational quantum algorithms (Cerezo et al., 2021) (VQE and standard QAOA) are affected by the barren plateau phenomenon (Wang et al., 2021), where the optimization landscape becomes increasingly flat, leading to vanishing gradients. This issue worsens as the parameter space scales linearly with the number of qubits while the ansatz's expressivity saturates exponentially, making optimization increasingly difficult. To mitigate this, we propose a Symmetric Arc Reduction technique to reduce the number of qubits required to execute a quantum circuit via the Quantum Approximate Optimization Algorithm (QAOA) ansatz.

The paper is structured as follows: Section 2 discusses relevant articles on quality assurance in domains related to vehicle routing. This effort aims to identify the research gaps that the review addresses. Background information on Quantum Approximate Optimization Algorithm (QAOA) and Quadratic Unconstrained Binary Optimization (QUBO) is covered in Section 3. The framework of our proposed Symmetric Arc Reduction (SAR) technique and the mathematical formulation of the Classical Vehicle Routing Problem are described in the methodology Section 4. The implication steps of our approach via QAOA are presented in section 5. The Time complexity and Results are briefly discussed in Sections 6 and 7, respectively. Finally, the conclusion of the paper is given in Section 8.

## 2 Literature review

### 2.1 Vehicle routing problem

The concept of the Vehicle Routing Problem was first introduced by Dantzig and Ramser as the Truck Dispatching Problem, which is a generalization of the Traveling Salesperson Problem (Dantzig and Ramser, 1959). The paper models the problem mathematically to minimize total travel cost and introduces a way to optimize how trucks are sent out to pick up and deliver goods. Clarke and Wright (1964) extended the study by considering multiple demands and multiple truck capacities, producing a near-optimal solution. Fischetti et al. (1997) devised a different non-spanning version of the Generalized Traveling Salesman Problem(GTSP), Equality-GTSP, and proposed an exact algorithm with a cutting plane and branch-and-bound approach. Pisinger and Ropke (2007) designed a heuristic, Adaptive Large Neighborhood Search (ALNS) algorithm to solve mathematically formulated variants of VRP as a rich pickup and delivery problem with time windows(RPDPTW). Liu et al. (2025) introduced a Fuzzy Logic Guided Genetic Algorithm (FLGA) to optimize routing costs such as time and distance. However, the authors clearly state the limitations of traditional evolutionary methods, which are prone to premature convergence, where the algorithm gets stuck in local optima because of rigid control parameters. To address this, the authors introduced additional Fuzzy Logic controllers to adjust crossover and mutation probabilities and cyclic repair operators to repair infeasible solutions.

Montané and Galvão (2006) tackled Vehicle Routing Problem with Simultaneous Pick-up and delivery(VRP-SPD). The authors developed a Tabu Search algorithm that uses different moves and the 2-opt technique to tweak routes within the same path and tested the approach on problem sizes ranging from 50 to 400 clients. Ai and Kachitvichyanukul (2009) introduced a generalized VRPSPD with an effective particle swarm optimization algorithm. Their approach utilized a random key-based representation and a multi-stage decoding process, demonstrating strong performance, competitiveness with existing methods, and the ability to find new best solutions when considering the fixed cost of vehicles. However, classical methods such as exact, heuristic, and meta-heuristic algorithms have been widely used to solve the vehicle routing problem (VRP); their performance tends to deteriorate as the problem size and complexity increase. As scalability increases, these approaches quickly become computationally intensive or fail to produce satisfactory solutions within a reasonable time frame (Mohanty et al., 2023). Conversely, quantum computing offers a compelling alternative by harnessing inherent parallelism from quantum phenomena such as superposition and entanglement Bennett et al. (2021).

### 2.2 Quantum vehicle routing

Optimization of the Vehicle Routing Problem through Quantum techniques typically begins with encoding the canonical QUBO formulation derived from the Lucas (2014) Ising model for the Traveling Salesman Problem. While this foundation quantifies primary routing constraints, recent research has focused on extending its structural richness to integrate real-world operational distribution requirements.

Lytrosyngounis and Lytrosyngounis (2025) proposed a hybrid quantum-classical strategy that combines quantum optimization with traditional machine learning techniques by utilizing IBM's Qiskit runtime. Testing on TSP examples with four to eight customers shows the hybrid approach reduces cost by 11.3%. Harwood et al. (2021) significantly addressed the challenge of solving the Vehicle Routing Problem with Time Windows(VRPTW) by developing and comparing various mathematical formulations, such as route-based and sequence-based models, and evaluating their complexity, demonstrating the potential benefits and challenges of applying quantum algorithms.

Bennett et al. (2021) pioneered an efficient algorithm for representing the solution space and implementing quantum walk operators tailored to CVRP instances. Their numerical simulations on 8 locations showed that the Quantum Walk-Based Optimization Algorithm(QWOA) could effectively amplify the probabilities of near-optimal solutions. Ariño Sales and Palacios Araos (2023) addressed that quantum clustering may outperform classical methods in cases of low clusterability or high randomness, common in real-world data. However, current hardware limitations challenge large-scale implementations. Syrichas and Crispin (2017) present a streamlined, energy-based parameter-tuning methodology for the Quantum Annealing (QA) approach called Fixed Quantum Annealing for capacitated vehicle routing problems (FJ-QACVRP), enabling effective large-scale problem solving with minimal tuning effort.

Mohanty et al. (2023) modeled a basic VRP solver for small instances (3 and 4 customers) using the variational quantum eigensolver, demonstrating the feasibility of quantum methods in practical applications. Feld et al. (2019) proposed and implemented a hybrid quantum-classical solution approach rooted in the classical 2-Phase heuristic clustering and Routing, which competes well with classical heuristics. Nonetheless, the approach leverages quantum computing for logistics optimization. Papalitsas et al. (2019) developed the explicitly stated QUBO formulation for the TSP with Time Windows(TSPTW). Their proposed method introduces auxiliary variables to relax inequality constraints into equalities, which are then squared in the Hamiltonian, yielding an effective yet computationally demanding approach that increases the number of binary decision variables. Further developing the line of research, Irie et al. (2019) established a comprehensive timetable QUBO model for a generalized Capacitated VRP (CVRP) with vehicle states and dynamic capacities. Although this framework enables advanced scheduling, it uses a large variable space to describe vehicle positions in discrete time intervals, resulting in repeated, excessive qubit overhead. Regarding fleet composition, Harikrishnakumar et al. (2020) have successfully applied Quantum Annealing to the Multi-Depot Capacitated VRP with heterogeneous vehicles. Beyond improving expressiveness, researchers have explored methods to enable scalable deployment. Clark et al. (2019) leveraged a D-Wave quantum annealer within a *qb-solv* classical decomposition as a hybrid quantum-classical approach for dynamic multi-robot routing to mitigate system-level bottlenecks such as embedding and communication latency. However, the intrinsic qubit and connectivity requirements of the QUBO model remain unoptimized. Finally, these contributions have extended the capabilities of quantum annealers to handle higher-complexity constraints, such as time windows, capacities, and heterogeneous fleets, while consistently imposing substantial qubit overhead. This overhead is a fundamental bottleneck for gate-model quantum algorithms where circuit width is a crucial restriction on near-term hardware.

To contextualize the resource efficiency and feasibility of the discussed methods, A detailed comparison of qubit requirements of various quantum VRP approaches is presented in Table 1. As observed, the majority of edge-based formulations scale quadratically, (≈*N*^2^), retaining the full graph structure. While dense encoding scales logarithmically, it often incurs high circuit-depth overhead due to complex decoding logic. Our proposed SAR-QAOA occupies and retains the shallow-circuit benefits of edge-based mapping while reducing the qubit requirement to exactly $\frac{n\left(n-1\right)}{2}$. Furthermore, by employing the Augmented Lagrangian Method (ALM), our approach avoids the ancillary qubit overhead typically associated with slack-variable constraint handling.

**Table 1:** Comparative analysis of quantum vehicle routing problem (VRP) approaches.

Reference	Methodology	Hardware	Qubits	QUBO variables	Model	Constraint handling
Mohanty et al. (2023)	Variational Quantum Eigensolver (VQE)	IBM Qiskit simulator	*n*(*n*−1)	*n*(*n*−1)	Gate-based	Penalty terms (slack)
Bennett et al. (2021)	Quantum Walk Optimization Algorithm (QWOA)	QuOp_MPI simulator	*N*log*N*	No QUBO formulation; direct index mapping	Gate-based	Index mapping
Lytrosyngounis and Lytrosyngounis (2025)	Hybrid QAOA/VQA with machine learning integration	IBM Sherbrooke simulator (127 qubits)	*n* ^2^	≈*n*^2^	Gate-based	Penalty terms (Slack)
Harwood et al. (2021)	QAOA and VQE	IBM Quantum QASM simulator	*n* ^2^	*n* ^2^	Gate-based	Penalty terms (slack)
Ariño Sales and Palacios Araos (2023)	Quantum annealing	D-wave advantage 6.1 via Amazon Braket	*n* ^2^	*n* ^2^	Annealing-based	Native annealing constraints
Syrichas and Crispin (2017)	Simulated quantum annealing	Classical CPU	None	Ising spins	Energy-based	Two-dimensional spin matrix
Feld et al. (2019)	Quantum annealing	D-wave 2000Q	*n* ^2^	*n* ^2^	Energy-based	Penalty terms (slack)
Papalitsas et al. (2019)	QUBO for TSPTW	D-Wave 2000Q	≥*n*^2^	*O*(*N*^3^+*NK*)	Annealing-based	Penalty terms (slack)
Irie et al. (2019)	Time-table QUBO	D-Wave 2000Q	≥*n*^2^	*O*(*TNKC*)	Annealing-based	Direct encoding and penalties
Harikrishnakumar et al. (2020)	Adiabatic quantum annealing	D-Wave 2000Q	≥*n*^2^	*O*(*NK*+Slack)	Annealing-based	Penalty terms (slack)
Clark et al. (2019)	Quantum Annealing	D-Wave 2000Q with *qbsolv*	≥*n*^2^	*O*(*RP*)	Annealing-based	Penalty terms (slack)
Proposed	Symmetric Arc Reduction via QAOA (SAR-QAOA)	Pennylane simulator (lightning.qubit)	$\frac{n\left(n-1\right)}{2}$	$\frac{n\left(n-1\right)}{2}$	Gate-based	UPM (zero ancilla)

### 2.3 Novelty and contributions

In this research, we develop a QUBO-oriented Symmetric Arc Reduction strategy(SAR) for the Vehicle Routing Problem that reduces the number of binary decision variables and, consequently, the qubit requirements for QAOA. The proposed SAR-QAOA technique is motivated by the symmetric edge representation explored in classical optimization Laporte (2024). To the best of our knowledge, this is the first study to employ this concept to construct a qubit-efficient QUBO model for quantum optimization of the VRP. This article primarily addresses how to map SARVRP onto a gate-based quantum computer and solve it using QAOA. Moreover, our method reduces the number of qubits for the VRP problem, based on edges as a path, from *n*(*n*−1) to half that. Figure 1 illustrates the overall workflow of this article.

**Figure 1:**
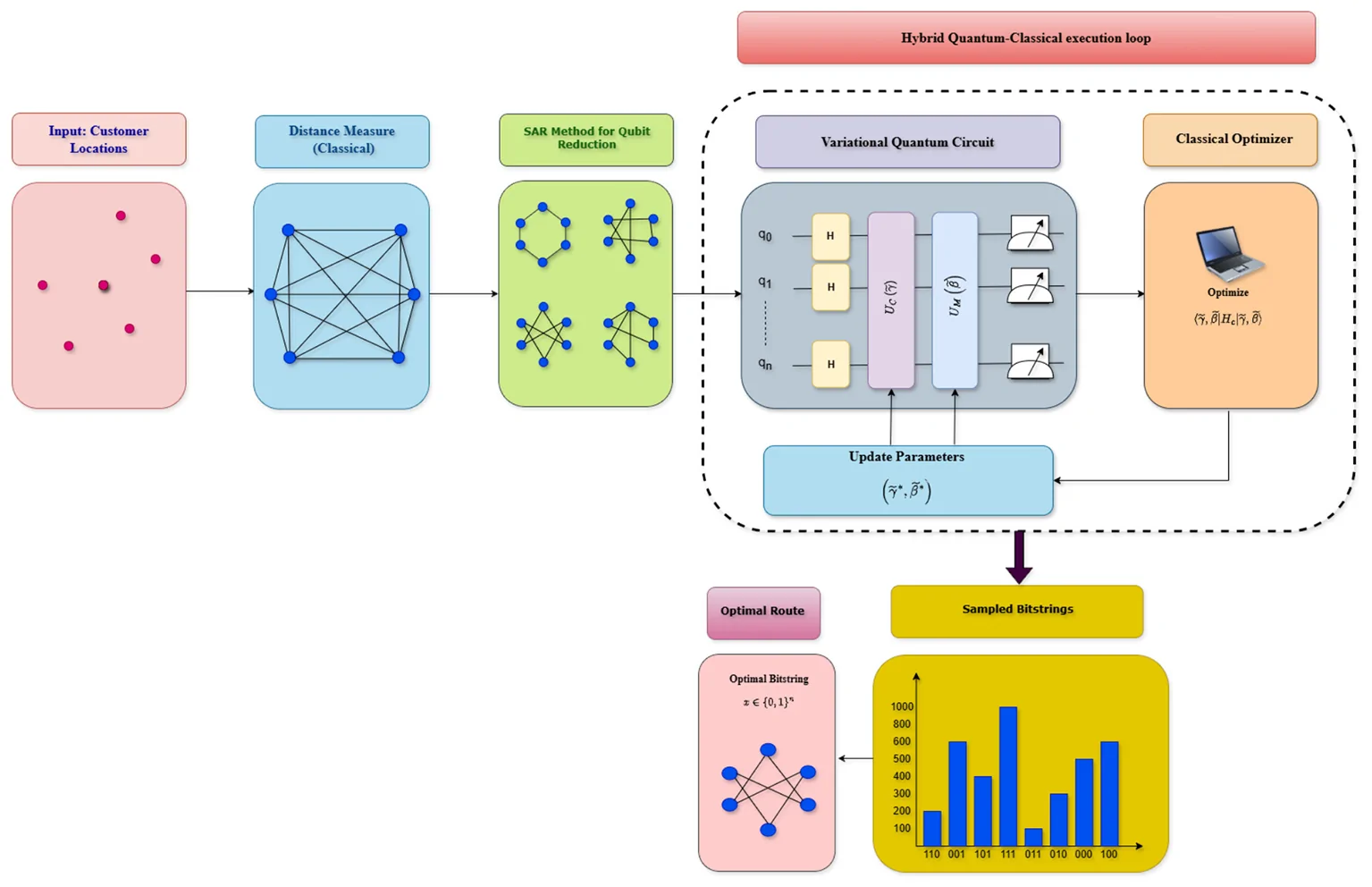
Proposed workflow.

The Key contributions are here:

1. In this study, we propose a Symmetric Arc Reduction technique (SAR) for reducing the number of binary decision variables through a binary integer programming formulation of the vehicle routing problem; that is, a graph pre-processing model that systematically detects and eliminates bidirectional non-optimal arcs. Unlike conventional distance-based pruning or dense full-graph formulations (Toth and Vigo, 2002) that retain *n*(*n*−1) variables, our SAR approach exploits symmetry to reduce the graph while preserving the feasible solution space, enabling a more efficient QUBO representation that is highly compatible with gate-based architectures.
2. We demonstrate a substantial decrease in resource overhead for binary edge formulations. whereas standard directed-graph mappings (Feld et al., 2019; Lucas, 2014; Azad et al., 2023) scale with *n*(*n*−1), the proposed SAR-QAOA framework solves instances with 4,5,6, and 7 nodes using only 6,10,15, and 21 qubits, respectively. This represents an approximate 50% reduction in qubit requirements compared with fully connected benchmarks, offering a better trade-off between qubit count and circuit depth than complex logarithmic encoding. However, no research has been found on edge-based VRP with qubit reduction.
3. By reducing the dimension, our proposed approach overcomes the connectivity and coherence constraints that render standard formulations intractable for *N* ≥ 5. We provide empirical evidence that SAR-QAOA consistently converges to valid routing solutions for 7-node instances (21 qubits). Conversely, standard QAOA formulations at this scale typically fail to produce feasible schedules because the search space explodes.

## 3 Background

### 3.1 Quantum Approximate Optimization Algorithm

The Quantum Approximate Optimization Algorithm (QAOA) is a variational hybrid algorithm proposed in 2014 by Farhi et al. (2014) to solve combinatorial optimization problems. The central idea behind QAOA is to combine quantum and classical computational techniques in a single iterative framework. Consider the minimization problem of an objective function (F(x)), where (*x*) represents an (*n*) -bit binary string, which can be equivalently formulated in terms of quantum Hamiltonian dynamics. Specifically, such a function (F) can be mapped onto a corresponding Hamiltonian $\left(H_{F}\right)$, whose eigenvalues correspond to the (2^*n*^) possible evaluations of (F(x)) over the entire domain. Consequently, the problem of determining the optimal value of (F) reduces to a particular instance of identifying the extremal (maximum or minimum) eigenvalues of the associated Hamiltonian $\left(\left(H_{F}\right)\right)$.

The core quantum circuit operation begins with initial state preparation, applying Hadamard gates **H** to all qubits ${\left(H|0\rangle \right)}^{⊗n}=|\psi_{0}\rangle =\frac{1}{\sqrt{2^{n}}}\left(|0\rangle +|1\rangle \right)$. This state prepares the system in a superposition of all *n* qubits, as implemented in Algorithm 1.

Algorithm 1 Quantum Approximate Optimization Algorithm (QAOA) 
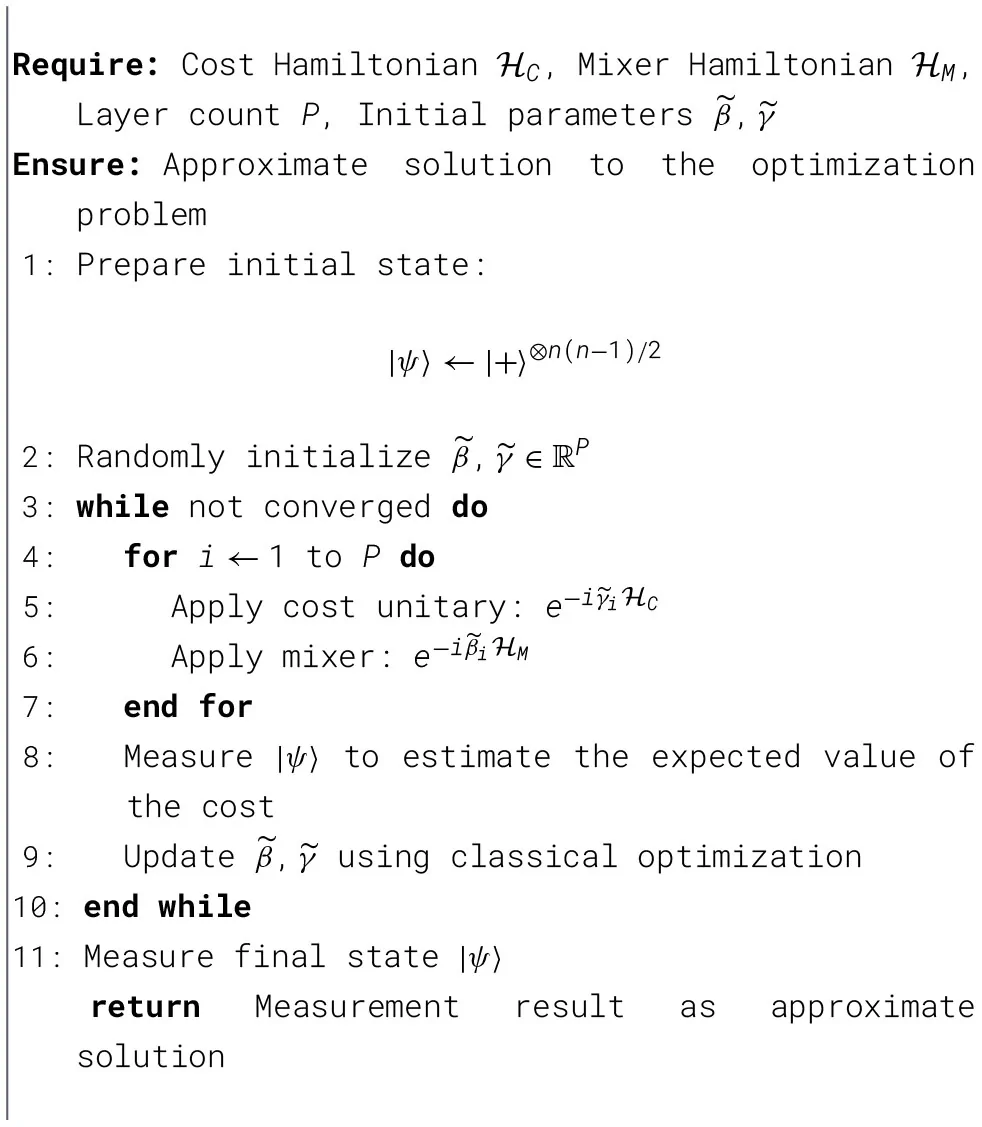

As depicted in Figure 2, the proposed SAR-QAOA circuit ansatz is constructed from a sequence of fundamental gates, where the Hadamard gate (*H*) initializes the system into an equal superposition state $|+\rangle^{⊗\frac{n\left(n-1\right)}{2}}$, enabling unbiased exploration of the solution space. The Rotation (*RZ*) gate encodes the linear terms of our CVRP cost function. The Ising Interaction (*RZZ*) gate implements the phase separator unitary; specifically, these gates encode the problem constraints and distance costs between nodes into the quantum state phases. Lastly, the Rotation (*RX*) gate enforces the mixing unitary, which promotes transitions between basis states, enabling the algorithm to escape local minima during the optimization process.

**Figure 2:**
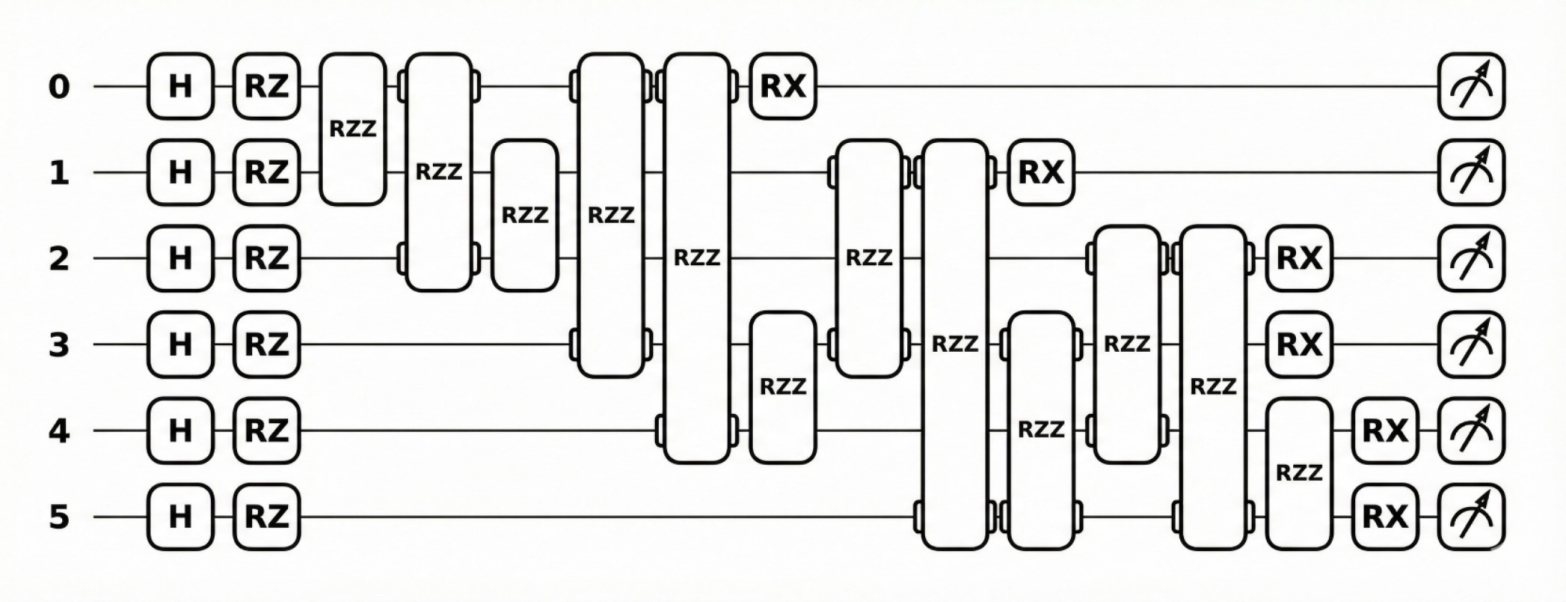
A Single-layer 4-customer (city) Quantum Circuit with six qubits by SAR-QAOA.

### 3.2 Quadratic unconstrained binary optimization

Quadratic Unconstrained Binary Optimization (Glover et al., 2019) is a mathematical model that is typically utilized for expressing a range of combinatorial optimization problems to be executed on quantum optimization algorithms. While “binary” denotes that the decision variables in this model can only have values of 0 or 1, “quadratic” describes the cost function's structure, which contains quadratic terms. The main goal of the QUBO is to transform a combinatorial optimization problem into a Hamiltonian, or total energy expression, within a quantum system. The general form of a QUBO (Quadratic Unconstrained Binary Optimization) problem can be written as:


$$\begin{array}{l} \underset{x\in {\left\{0,1\right\}}^{n}}{\min}x^{⊤}Qx \end{array}$$ (1)

In essence, the paper presents a comprehensive framework for translating classical optimization problems into quantum optimization problems, with the overarching goal of advancing the QAOA ansatz as a potentially powerful computational paradigm. The following equations demonstrate how the objective function of classical NP problems is translated into quantum optimization problems through Ising models (Lucas, 2014), as a linear combination of Hamiltonians, each scaled by a real-valued coefficient.

## 4 Methodology

To detail our approach, we structure the methodology around five critical phases, moving logically from problem formulation to quantum execution. We begin by introducing the Vehicle Routing Problem (VRP) and detailing its mathematical formulation. This groundwork is immediately followed by a deep dive into Symmetric Arc Reduction(SAR) techniques, crucial methods that significantly reduce VRP complexity by employing a spanning subgraph approach. The core of our quantum mapping is then presented, where the cost function is defined, and the adopted encoding strategies, including SAR-based edge encoding) are outlined for translating the problem onto a quantum device. Following the encoding, we meticulously detail the implementation of the Quantum Approximate Optimization Algorithm (QAOA) framework. The final section concludes the methodology by describing the complete SAR procedure for constructing the reduced QUBO formulation and its corresponding QAOA circuit for quantum optimization.

### 4.1 Vehicle Routing Problem

The Vehicle Routing Problem (VRP) is a well-studied NP-hard combinatorial optimization challenge that involves designing efficient routes for a fleet of k vehicles to serve n-1 customers (Lenstra and Kan, 1981). Each route begins and ends at the central depot, ensuring each customer is visited once while minimizing total travel distance. Route optimization for a uniform fleet of vehicles with defined capacity constraints is the main focus of the Link-based Capacitated Vehicle Routing Problem (CVRP) (Azad et al., 2023), a special variant of this topic. Each vehicle must deliver goods to customers with predetermined demands, starting and returning to the depot, with the objective of minimizing overall travel cost or time while adhering to demand and capacity constraints. Let N={0,1,2,…,n-1} denote the set of customer nodes, where the node 0 be the depot. The delivery process is executed by a set of vehicles or trucks $V_{s}=\left\{1,2,\ldots ,k\right\}$. Let us define the binary variables *x*_*ij*_ are defined in Equation 2 as:


$$x_{ij}=\{\begin{array}{ll} 1, & \text{if a vehicle traverses from node }i\text{ to }j\\ 0, & \text{otherwise} \end{array}$$ (2)

For simplicity, customer demand is fulfilled instantaneously upon the vehicle's arrival at the designated node. The cost of travel between two nodes *i* and *j* is denoted by $C_{ij}$, and the primary optimization objective (Equation 3) is to minimize the aggregate distance associated with all traversed links. The problem can be expressed as:


$$\begin{array}{l} \min\underset{i}{\sum }\underset{j}{\sum }C_{ij}x_{ij},\;\forall i,j\in N, \end{array}$$ (3)

subject to:


$$\begin{array}{l} \underset{j>0}{\sum }x_{ij}=1,\;\forall i>0, \end{array}$$ (4)

$$\begin{array}{l} \underset{j}{\sum }x_{ij}=\underset{j}{\sum }x_{ji},\;\forall i, \end{array}$$ (5)

$$\begin{array}{l} \underset{j}{\sum }x_{0j}=k,\;\underset{i}{\sum }x_{i0}=k, \end{array}$$ (6)

$$\begin{array}{l} \underset{i\in S}{\sum }\underset{j\notin S}{\sum }x_{ij}\geq 1,\;\forall S\subset N,\;2\leq |S|\leq n-1. \end{array}$$ (7)

$$x_{ij}\in \{0,1\},\;\forall i,j\in N$$ (8)

Constraint Equation 4 establishes a fundamental requirement: each customer must be visited exactly once. To maintain logical travel paths, Constraint Equation 5 imposes flow conservation at every node, ensuring that if a vehicle enters a node, it also leaves it. Furthermore, the restriction Equation 6 explicitly controls the fleet size, specifying that precisely *k* vehicles must both depart from and return to the central depot. vehicles depart and return to the depot. Finally, as a subtour elimination condition, Constraint Equation 7 addresses possible invalid solutions, preventing disconnected cycles and ultimately guaranteeing overall route connectivity back to the depot. Constraint Equation 8 uses binary decision variables.

### 4.2 Motivation

Our proposed study is inspired by the resource gap between classical optimization frameworks and the constraints of current quantum computing platforms. Although classical Integer programming models (Laporte, 2024) can model CVRP effectively, translating them into quantum models incurs significant qubit overhead. Specifically, representing the integer-valued depot edges (*x*_0*j*_ ∈ 0, 1, 2) through standard binary necessitates, it requires $\frac{n^{2}+3n}{2}$ qubits. Given the limited coherence and gate fidelities of Noisy Intermediate-Scale Quantum devices, this quadratic qubit expansion becomes a formidable barrier to scalability. To address this, our proposed SAR technique reformulated the VRP as a Binary Integer Programming (BIP) problem. By encoding into a binary subspace, our framework reduces the qubit requirement from $\frac{n\left(n-1\right)}{2}$, streamlining a more resource-efficient QUBO formulation. This reduction decreases the resource requirements of the quantum optimization model and facilitates its implementation on quantum simulators, while providing a foundation for subsequent research on emerging quantum hardware.

### 4.3 Framework

The Symmetric Arc Reduction (SAR) approach improves model efficiency and reduces computational complexity by leveraging symmetry in undirected distance graphs. In our model, the graph is effectively undirected (that is *v*_*i*_→*v*_*j*_ is identical to *v*_*j*_→*v*_*i*_), making the representation of both directions redundant. The SAR technique removes this redundancy by treating the problem as undirected. Consequently, the number of variables required dips from *n*(*n*−1)(for a directed model) to $\frac{n\left(n-1\right)}{2}$, adequately halving the problem size. This dimensionality reduction improves the scalability and computational efficiency of classical and quantum optimization frameworks.

### 4.4 Advantages

The SAR method offers key advantages that enhance both classical and quantum optimization frameworks. Most immediately, it halves the problem size. Crucially, it achieves this efficiency without compromising the search space; because it eliminates only redundant arcs, the feasible solution space remains intact. For quantum applications, this dimensional reduction is particularly valuable: it directly lowers the qubit requirement in QUBO formulations, which is essential for testing larger problem instances on current near-term quantum devices. Furthermore, the simplified graph structure streamlines implementing the edge-based encodings required for hybrid classical-quantum algorithms such as the Quantum Approximate Optimization Algorithm (QAOA).

### 4.5 Assumptions

The Novel SAR approach to the classical VRP requires several standard assumptions (Dantzig and Ramser, 1959; Laporte, 1992) to ensure that the resulting optimization framework is both well-defined and computationally tractable.

1. All vehicles start and end their routes at a single central depot.
2. Customer locations and demands are known and remain fixed.
3. Each customer demand is known in advance; randomness is not considered.
4. Every vehicle has a fixed load limit that cannot be exceeded.
5. The costs of travel or distances between two locations are positive and symmetric (that is, (*c*_*ij*_ = *c*_*ji*_ ≥ 0)).
6. Each customer is visited exactly once by a single vehicle.
7. Vehicles serve customers one at a time without overlap.
8. All vehicles are identical in capacity and cost.
9. There are no direction-dependent constraints, such as time windows, priority deliveries, one-way route constraints, and asymmetric service costs.

To apply the SAR method and reduce the number of binary variables, we assume that the cost matrix is symmetric (i.e., (*c*_*ij*_ = *c*_*ji*_)) and that the graph structure is static and undirected. We also do not consider direction-dependent constraints such as time windows or one-way travel. These assumptions allow us to exploit graph symmetry, reduce redundant arcs, and map unordered node pairs to unique QUBO indices efficiently.

### 4.6 Symmetric Arc Reduction

Let G=(V,E) be a complete directed graph with node set V={0,1,…,n-1} and |V|=n, where node 0 represents the depot and C=V\{0} denotes the customer set. Let the cost matrix satisfy the symmetry condition $c_{ij}=c_{ji},\;\forall i,j\in N,i\neq j$. Define the binary arc variables *y*_*ij*_ = 1, if arc (*i, j*) is used, 0 otherwise. The classical directed VRP objective function of Equation 3 is $min\underset{i\neq j}{\sum }C_{ij}y_{ij}$. Routes are simple cycles that start and end at the depot. Let us define the undirected edge set E={(i,j)⊂V|i<j}. Define the binary edge variables *x*_*ij*_ = 1, if edge (*i, j*) is used, 0 otherwise. Next, we define the transformation mapping. Let $Y=\left\{y\in {\left\{0,1\right\}}^{n\left(n-1\right)}:\text{y satisfies directed VRP}\right\}$ and Let $X=\left\{x\in {\left\{0,1\right\}}^{\frac{n\left(n-1\right)}{2}}:\text{x satisfies undirected VRP}\right\}$. Now we define a function, Φ:Y→X, such that Φ(*y*)_*ij*_: = *y*_*ij*_+*y*_*ji*_, *i*<*j*. Since routes are simple cycles, atmost one of *y*_*ij*_, *y*_*ji*_ equals 1, hence *x*_*ij*_ ∈ {0, 1}. This reduces the number of decision variables from *n*(*n*−1) (Azad et al., 2023) to $\frac{n\left(n-1\right)}{2}$ is shown in Figure 3. Define $\alpha_{ij}=\{\begin{array}{ll} 2, & \text{if }i=0\text{ or }j=0\\ 1, & \text{otherwise} \end{array}$ represents the arc weight factor, which emphasizes a single edge in the objective function. The Proposed approach is presented in Algorithm 2.

**Figure 3:**
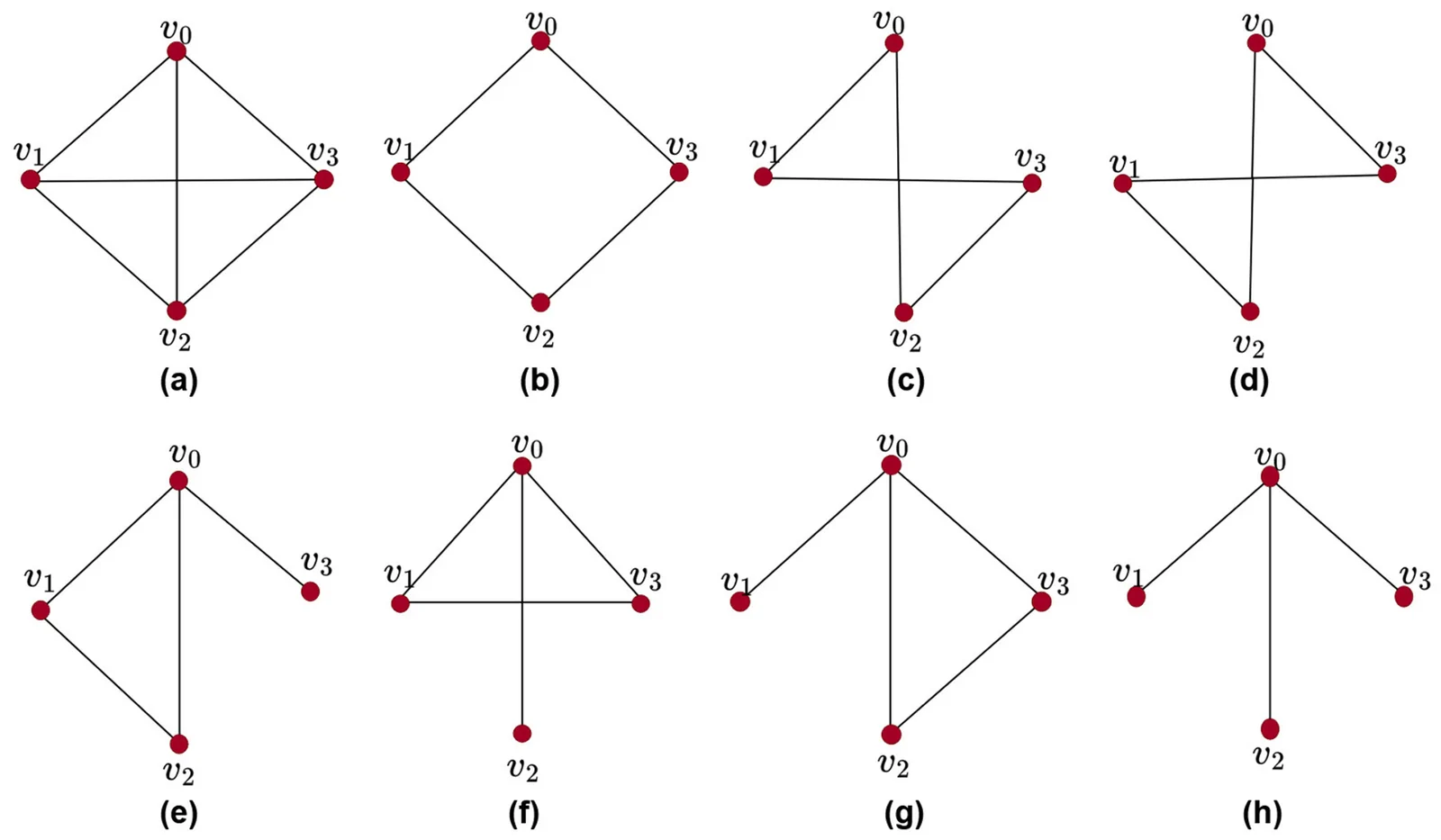
**(a)** *K*_4_ complete graph, By our SAR approach **(b–h)** are valid sub graphs with fixed vertex *v*_0_ of *K*_4_, where **(b, c, d)** for *n* = 4, *k* = 1, **(e, f, g)** for *n* = 4, *k* = 2, and **(h)** for *n* = 4, *k* = 3, respectively.

Algorithm 2 A qubit-efficient SAR-QAOA-based approach. 
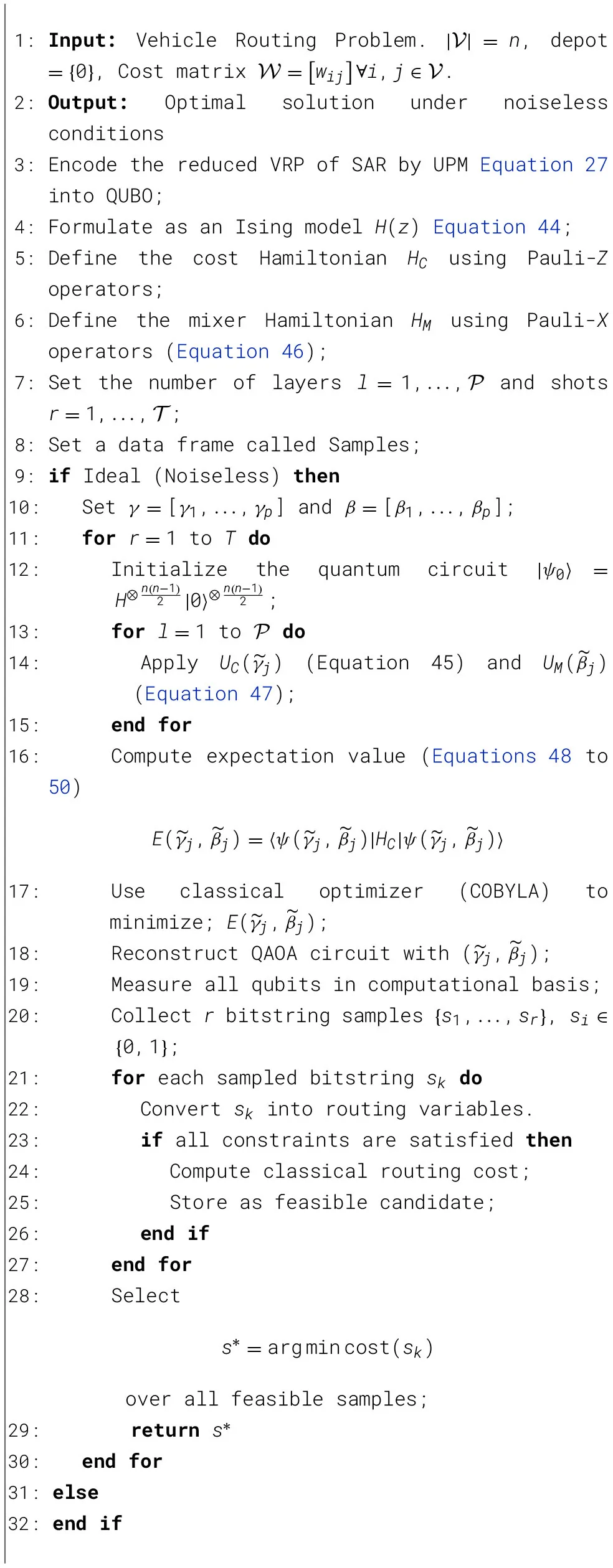

** Theorem 1**. For any feasible directed solution *y*, the objective value of the directed formulation equals the objective value of the SAR formulation


$$\begin{array}{l} \underset{i\neq j}{\sum }C_{ij}y_{ij}=\underset{i<j}{\sum }\alpha_{ij}C_{ij}x_{ij} \end{array}$$ (9)

*Proof:* Partition the directed objective Equation 9 into two components:


$$\begin{array}{l} \underset{i\neq j}{\sum }c_{ij}y_{ij}=\underset{i\neq j\begin{array}{c} i,j\in C \end{array}}{\sum }c_{ij}y_{ij}+\underset{j\in C}{\sum }\left(c_{0j}y_{0j}+c_{j0}y_{j0}\right) \end{array}$$ (10)

Since routes are simple cycles, each customer-customer undirected edge appears at most once, and each of *y*_*ij*_ or *y*_*ji*_ equals 1 if edge *i, j* is used. Using symmetry *c*_*ij*_ = *c*_*ji*_, Thus


$$\begin{array}{l} \underset{\begin{array}{c} i\neq j\\ i,j\in C \end{array}}{\sum }c_{ij}y_{ij}=\underset{\begin{array}{c} i<j\\ i,j\in C \end{array}}{\sum }c_{ij}x_{ij}. \end{array}$$ (11)

Each vehicle route starts and ends at depot 0. For every customer *j* served directly by depot, exactly two directed arcs exist, that is (0, *j*)and(*j*, 0). Thus,


$$\begin{array}{l} c_{0j}y_{0j}+c_{j0}y_{j0}=c_{0j}+c_{0j}=2c_{0j} \end{array}$$ (12)

$$\begin{array}{l} x_{0j}=y_{0j}+y_{j0}=1 \end{array}$$ (13)

$$\begin{array}{l} c_{0j}y_{0j}+c_{j0}y_{j0}=2c_{0j}x_{0j}. \end{array}$$ (14)

From Equations 12–14, summing over all depot-adjacent edges, we have


$$\begin{array}{l} \underset{j\in C}{\sum }\left(c_{0j}y_{0j}+c_{j0}y_{j0}\right)=\underset{j\in C}{\sum }2c_{0j}x_{0j} \end{array}$$ (15)

By combining Equations 11, 15 and substituting into Equation 10 we get Equation 16 as follows:


$$\begin{array}{l} \underset{i\neq j}{\sum }c_{ij}y_{ij}=\underset{\begin{array}{c} i<j\\ i,j\in C \end{array}}{\sum }c_{ij}x_{ij}+\underset{j\in C}{\sum }2c_{0j}x_{0j} \end{array}$$ (16)

This equals Equation 17, as


$$\begin{array}{l} \underset{i<j}{\sum }\alpha_{ij}c_{ij}x_{ij} \end{array}$$ (17)

Where $\alpha_{ij}=\{\begin{array}{ll} 2, & \text{if }i=0\text{ or }j=0\\ 1, & \text{otherwise} \end{array}$. Under the mapping Φ:Y→X, such that Φ(*y*)_*ij*_: = *y*_*ij*_ + *y*_*ji*_, *i* < *j*, the constraint correspondence of the directed degree constraints in Equations 5–7 become the undirected degree conditions in Equations 18–20, respectively.


$$\begin{array}{l} \underset{i<k}{\sum }x_{ik}+\underset{k<j}{\sum }x_{kj}=2,\;\forall k\in C\backslash \left\{0\right\} \end{array}$$ (18)

$$\begin{array}{l} \underset{j\in C}{\sum }x_{0j}=2k \end{array}$$ (19)

Subtour elimination constraints


$$\begin{array}{l} \underset{i\in S}{\sum }\underset{j\notin S}{\sum }x_{ij}\geq 2,\;\forall S\subset C \end{array}$$ (20)

Thus feasibility is preserved.

### 4.7 Formulation of VRP using Symmetric Arc Reduction (SAR)

The Symmetric Arc Reduction (SAR) method transforms a symmetric directed VRP into an undirected edge-based formulation with weighted depot edges, preserving objective and feasible-solution equivalence. Moreover, we use the structure of the deterministic symmetric vehicle routing problem to reduce variables by eliminating redundant directed variables through projection. In the binary undirected model, each route must connect the depot to two different customers or nodes. The formulation of our proposed SAR as a Binary Integer Programming (BIP) model can be written as follows:


$$\begin{array}{l} F\left(x\right)=\text{Minimize}\underset{i<j}{\sum }\alpha_{ij}w_{ij}x_{ij} \end{array}$$ (21)

subject to


$$\begin{array}{l} \;m\leq \overset{n-1}{\underset{j=1}{\sum }}x_{0j}\leq n-1, \end{array}$$ (22)

$$\begin{array}{l} 1\leq \left(\underset{i<j}{\sum }x_{ij}+\underset{j<k}{\sum }x_{jk}\right)\leq 2,\;\forall j\in V\backslash \left\{0\right\}, \end{array}$$ (23)

$$\begin{array}{l} \left(\underset{i<j}{\sum }x_{ij}+\underset{j<k}{\sum }x_{jk}\right)+2x_{0j}\geq 2,\;\forall j\in V\backslash \left\{0\right\}, \end{array}$$ (24)

$$\begin{array}{l} \underset{i\in S}{\sum }\underset{j\notin S}{\sum }x_{ij}\geq 2,\;\forall S\subset V,\;2\leq |S|\leq n-1. \end{array}$$ (25)

$$\begin{array}{l} x_{ij}\in \left\{0,1\right\},\;\forall i,j\in V,i<j. \end{array}$$ (26)

Equation 21 defines the objective of minimizing the total travel cost. In Constraint Equation 22 where *m* = min(*n*−1, *k*+1), Depot degree constraint regulates the number of vehicles(*k*) leaving the depot. Constraint Equation 23 (Customer degree constraint) enforces that each customer is visited exactly once by ensuring degree two at each customer node. Constraint Equation 24 (Depot–customer connectivity) guarantees connectivity between depot and customers. Finally, constraint Equation 25 (Subtour elimination) eliminates infeasible subtours, where Equation 26 specifies binary decision variables. From Tables 2, 3, the General Feasible solutions of problem Equations 21–26 is shown with eight nodes upto seven vehicles for convenience.

**Table 2:** Subgraphs with *n* nodes and *k* vehicles.

n/k	1	2	3	4	5	6	7
3	$\frac{\left(n-1\right)!}{2}$	1	***	***	***	***	***
4	$\frac{\left(n-1\right)!}{2}$	$\frac{\left(n-1\right)!}{2}$	1	***	***	***	***
5	$\frac{\left(n-1\right)!}{2}$	$\frac{5\left(n-1\right)!}{8}$	$\frac{\left(n-1\right)!}{4}$	1	***	***	***
6	$\frac{\left(n-1\right)!}{2}$	$\frac{3\left(n-1\right)!}{4}$	$\frac{3\left(n-1\right)!}{8}$	$\frac{\left(n-1\right)!}{12}$	1	***	***
7	$\frac{\left(n-1\right)!}{2}$	$\frac{7\left(n-1\right)!}{8}$	$\frac{25\left(n-1\right)!}{48}$	$\frac{5\left(n-1\right)!}{24}$	$\frac{\left(n-1\right)!}{48}$	1	***
8	$\frac{\left(n-1\right)!}{2}$	(*n*−1)!	$\frac{5\left(n-1\right)!}{8}$	$\frac{3\left(n-1\right)!}{16}$	$\frac{\left(n-1\right)!}{24}$	$\frac{\left(n-1\right)!}{120}$	1

***, not applicable.

**Table 3:** Total number of subgraphs for SAR approach up to *n* = 8 nodes.

Nodes(n)	Subgraphs(S)
3	$1+\frac{\left(n-1\right)!}{2}$
4	1+(*n*−1)!
5	$1+\frac{7\left(n-1\right)!}{8}$
6	$1+\frac{41\left(n-1\right)!}{24}$
7	$1+\frac{17\left(n-1\right)!}{8}$
8	$1+\frac{189\left(n-1\right)!}{80}$

## 5 Steps for QAOA ansatz for the routing problem under SAR method

### 5.1 Implications for quantum optimization

In edge-based QUBO formulations, each arc corresponds to a qubit. The SAR method minimizes the required number of qubits while retaining all structural properties of the problem. This makes the approach particularly suitable for implementation on quantum annealer and gate-based devices, where qubit resources are limited.

### 5.2 The unbalanced penalization method

The unbalanced penalization method (UPM) (Montaez-Barrera et al., 2024) restructures a constrained binary optimization problem into a QUBO without integrating auxiliary slack variables. As an alternative to converting inequality constraints into equalities, each constraint is internalized asymmetrically within the objective through linear $\left(A_{1}\right)$ and quadratic penalty $\left(A_{2}\right)$ components. This approach retains the original decision space and avoids increasing the variable set, which is especially beneficial for quantum implementations where the number of binary variables significantly influences qubit requirements. Consider a simple three decision variables (*n* = 3); $\underset{\begin{array}{c} x\in {\left\{0,1\right\}}^{3} \end{array}}{\min}5x_{1}+3x_{2}+2x_{3}$subject to 2*x*_1_ + *x*_2_ + *x*_3_ ≤ 2. The QUBO model of the toy example using UPM is,


$$\begin{array}{r} \underset{\begin{array}{c} x\in {\left\{0,1\right\}}^{3} \end{array}}{\min}5x_{1}+3x_{2}+2x_{3}-A_{1}\left[2x_{1}+x_{2}+x_{3}-2\right]\\ +A_{2}\left[{\left(2x_{1}+x_{2}+x_{3}-2\right)}^{2}\right] \end{array}$$

Finally, the compact QUBO form can be written as,


$$\begin{array}{c} f\left(x\right)=\left[\begin{array}{ccc} x_{1} & x_{2} & x_{3} \end{array}\right]\\ \left[\begin{array}{ccc} 5-2A_{1}-4A_{2} & 4A_{2} & 4A_{2}\\ 0 & 3-A_{1}-3A_{2} & 2A_{2}\\ 0 & 0 & 2-2A_{1}-3A_{2} \end{array}\right]\\ \left[\begin{array}{c} x_{1}\\ x_{2}\\ x_{3} \end{array}\right]+2A_{1}+4A_{2}. \end{array}$$

The cost Hamiltonian *H*(*z*) when $A_{1}=A_{2}=1$ is computed by replacing the elements of the matrix Q in Equation 1. Therefore,


$$\begin{array}{l} H\left(z\right)=z_{1}z_{2}+z_{1}z_{3}+0.5z_{2}z_{3}-1.5z_{1}-2.5z_{2}-1.5z_{3}+6. \end{array}$$

### 5.3 Mapping to a quantum computer

In this study, we utilize a Quadratic Unconstrained Binary Optimization (QUBO) mapping in which each edge is represented by a single qubit. Accordingly, the Symmetric Arc Reduction (SAR) method requires only $\frac{n\left(n-1\right)}{2}$ qubits in total. Moreover, to comply with constraints, traditional QUBO formulations often rely on penalty-driven strategies. Although effective, these techniques substantially increase the number of qubits used because inequality constraints must be reinterpreted as equality constraints by adding slack variables. Quantitatively, converting an inequality constraint *C* into an equality requires adding a slack variable encoded by *log*_2_(*C*+1) ancillary qubits. Moreover, for CVRP of *k* vehicles, this introduces an overhead of k*log_2_(*C*+1) qubits. This overhead is significant given the limited qubit availability of current NISQ devices. To address this, we incorporate an Unbalanced Penalization Method (UPM), which provides a more efficient way to embed constraints into the QUBO framework. Within this study, we apply this methodology to reformulate the SAR-based Vehicle Routing Problem (VRP) into a QUBO representation of the following form:


$$\begin{array}{l} {ℋ}_{cost}(x)=C(x)+A_{1}\{\left(\sum_{j=1}^{n-1}x_{oj}-m\right)+\left((n-1)-\sum_{j=1}^{n-1}x_{oj}\right)\\ +\left(\sum_{i<j}x_{ij}+\sum_{j<k}x_{jk}-1\right)+\left(2-\sum_{i<j}x_{ij}+\sum_{j<k}x_{jk}\right)\\ +\left(\sum_{i<j}x_{ij}+\sum_{j<k}x_{jk}+2x_{oj}-2\right)+\left(\sum_{i\in S}\sum_{j\notin S}x_{ij}-2\right)\}\\ +A_{2}\{{\left(\sum_{j=1}^{n-1}x_{oj}-m\right)}^{2}+{\left((n-1)-\sum_{j=1}^{n-1}x_{oj}\right)}^{2}\\ +{\left(\sum_{i<j}x_{ij}+\sum_{j<k}x_{jk}-1\right)}^{2}+{\left(2-\sum_{i<j}x_{ij}+\sum_{j<k}x_{jk}\right)}^{2}\\ +{\left(\sum_{i<j}x_{ij}+\sum_{j<k}x_{jk}+2x_{oj}-2\right)}^{2}\\ +{\left(\sum_{i\in S}\sum_{j\notin S}x_{ij}-2\right)}^{2}\} \end{array}$$ (27)

where the penalty parameters $A_{1}$ and $A_{2}$ ≥ 0, in Equation 27 must be chosen sufficiently large to dominate any decrease in the original objective function that could be achieved by transitioning to an infeasible solution (Ayodele, 2022). Therefore, we set the penalty ∀*l* ∈ {1, 2}, $A_{l}>A_{UB}$, where $A_{UB}=\underset{i,j}{\sum }w_{ij}$. The Unbalanced Penalization Method (UPM) generally requires fewer qubits than the slack variable approach because it embeds constraints directly into the objective function through penalty terms. Conversely, the slack variable method typically introduces an auxiliary variable for each constraint to ensure feasibility. The next step is to convert this Equation 27 formulation to an Ising problem by converting the cost function to an Ising Hamiltonian through a change of variables (Equation 28) defined as:


$$\begin{array}{l} x_{ij}=\frac{1-Z_{ij}}{2},Z_{ij}\in \left\{-1,1\right\} \end{array}$$ (28)

Where $Z_{ij}=\left[\begin{array}{cc} 1 & 0\\ 0 & -1 \end{array}\right]$ is the PauliZ operator. This promotes our cost function $H_{cost}\left(x\right)$ into an operator ${Ĥ}_{C}$.

### 5.4 Penalty parameter selection

The selection of the penalty coefficient is critical to preserving the equivalence between the original constrained optimization problem and its unconstrained QUBO reformulation. An insufficient penalty coefficient may permit infeasible solutions to attain a lower QUBO objective value than feasible solutions. The following theorem establishes a sufficient condition for selecting the penalty coefficient.

** Theorem 2 (Sufficient penalty condition.)**. *Let the proposed edge-based SAR-QUBO formulation (Equation 29) be*


$$\begin{array}{l} H\left(\text{x}\right)=C\left(\text{x}\right)+\rho P\left(\text{x}\right), \end{array}$$ (29)

where


$$\begin{array}{l} C\left(\text{x}\right)=\underset{i<j}{\sum }w_{ij}x_{ij} \end{array}$$ (30)

*Equation 30 denotes the routing objective*, *P*(**x**) ≥ 0 *is the aggregate penalty function representing all routing constraints, and*


$$\begin{array}{l} P\left(\text{x}\right)=0 \end{array}$$ (31)

*if and only if* **x** *satisfies all routing constraints, as given in Equation 31*.

*Let F and I denote the sets of feasible and infeasible solutions, respectively, and define*


$$\begin{array}{l} \text{Δ}C=\underset{{\text{x}}_{F}\in \text{F},{\text{x}}_{I}\in \text{I}}{\max}\left(C\left({\text{x}}_{F}\right)-C\left({\text{x}}_{I}\right)\right), \end{array}$$ (32)

*Equation 32 represents the maximum reduction in routing cost obtainable by violating one or more routing constraints*.

*Furthermore, let*


$$\begin{array}{l} \delta =\underset{\text{x}\in \text{I}}{\min}P\left(\text{x}\right),\;\delta >0, \end{array}$$ (33)

*Here, Equation 33 denotes the minimum positive penalty associated with an infeasible solution*.

*If*


$$\begin{array}{l} \rho >\frac{\text{Δ}C}{\delta }, \end{array}$$ (34)

*then from Equation 34, every global minimizer of the QUBO objective satisfies all routing constraints and therefore corresponds to an optimal solution of the original constrained optimization problem*.

*Proof:* Let **x**_*F*_ ∈ F be any feasible solution. Since *P*(**x**_*F*_) = 0, its corresponding QUBO objective Equation 35 is


$$\begin{array}{l} H\left({\text{x}}_{F}\right)=C\left({\text{x}}_{F}\right). \end{array}$$ (35)

Now consider any infeasible solution **x**_*I*_ ∈ I. By definition of δ,


$$\begin{array}{l} P\left({\text{x}}_{I}\right)\geq \delta . \end{array}$$ (36)

$$\begin{array}{l} \begin{array}{cc} H\left({\text{x}}_{I}\right) & =C\left({\text{x}}_{I}\right)+\rho P\left({\text{x}}_{I}\right)\\ \geq C\left({\text{x}}_{I}\right)+\rho \delta . \end{array} \end{array}$$ (37)

From the definition of Δ*C*,


$$\begin{array}{l} C\left({\text{x}}_{I}\right)\geq C\left({\text{x}}_{F}\right)-\text{Δ}C. \end{array}$$ (38)

Substituting the above inequality (from Equations 36–38) into the QUBO objective yields


$$\begin{array}{l} \begin{array}{cc} H\left({\text{x}}_{I}\right) & \geq C\left({\text{x}}_{F}\right)-\text{Δ}C+\rho \delta . \end{array} \end{array}$$ (39)

If


$$\begin{array}{l} \rho >\frac{\text{Δ}C}{\delta }, \end{array}$$ (40)

then


$$\begin{array}{l} \rho \delta >\text{Δ}C, \end{array}$$ (41)

which implies


$$\begin{array}{l} H\left({\text{x}}_{I}\right)>C\left({\text{x}}_{F}\right)=H\left({\text{x}}_{F}\right). \end{array}$$ (42)

Hence from (Equations 39–42), every infeasible solution has a strictly larger QUBO objective value than every feasible solution. Consequently, no infeasible solution can be a global minimizer.

Since the QUBO objective coincides with the original routing objective for every feasible solution,


$$\begin{array}{l} H\left(\text{x}\right)=C\left(\text{x}\right),\;\forall \text{x}\in \text{F}, \end{array}$$ (43)

Minimizing the proposed QUBO formulation is equivalent to minimizing the original constrained optimization problem. Therefore, every global minimizer of the proposed QUBO satisfies all routing constraints.

### 5.5 Cost Hamiltonian: problem encoding

The Cost Hamiltonian ${Ĥ}_{C}$, encodes the objective functions and it is given by:


$$\begin{array}{l} {Ĥ}_{C}=cĨ+\overset{N}{\underset{j=1}{\sum }}a_{j}{\tilde{P}}_{Z_{j}}+\overset{N}{\underset{j=1}{\sum }}\overset{N}{\underset{k=1}{\sum }}b_{jk}{\tilde{P}}_{Z_{j}Z_{k}} \end{array}$$ (44)

where:

• *c, a*_*j*_ & *b*_*jk*_ are real-valued coefficients.
• $\overset{N}{\underset{j=1}{\sum }}a_{j}{\tilde{P}}_{Z_{j}}$ represents linear terms.
• $\overset{N}{\underset{j=1}{\sum }}\overset{N}{\underset{k=1}{\sum }}b_{jk}{\tilde{P}}_{Z_{j}Z_{k}}$ accounts for pairwise (quadratic) interactions.

The goal is to find the ground state of ${Ĥ}_{C}$, which corresponds to the optimal solution. Trotterization approximates the cost operator as:


$$\begin{array}{r} U_{C}\left(\tilde{\gamma_{j}}\right)=e^{-i{\tilde{\gamma_{j}}}_{j}{ℋ}_{C}}\approx \left(e^{-i\tilde{\gamma_{j}}c\tilde{I}}\right)\\ \left(\prod_{k=1}^{k=N}e^{-i\tilde{\gamma_{j}}a_{k}{\tilde{P}}_{zk}}\right)\left(\prod_{k=1}^{k=N}\prod_{m=1}^{m=N}e^{-i\tilde{\gamma_{j}}b_{km}{\tilde{P}}_{zkzm}}\right) \end{array}$$ (45)

### 5.6 Mixer Hamiltonian

The Mixer Hamiltonian ${Ĥ}_{M}$ encourages the system to explore different possible states. It typically takes the form of the Pauli-X operator $\left(\left[\begin{array}{cc} 0 & 1\\ 1 & 0 \end{array}\right]\right)$:


$${\hat{ℋ}}_{M}=\sum_{k=1}^{k=N}{\tilde{P}}_{Z_{X_{k}}}$$ (46)

where ${\tilde{P}}_{Z_{X_{k}}}$ is a Pauli-X operator located in the *k*^*th*^ position, while the rest of the operators are identity gates. The evolution under the Mixer Hamiltonian is discretized as,


$$\begin{array}{l} U_{M}\left({\tilde{\beta }}_{j}\right)=e^{-i{\tilde{\beta }}_{j}{Ĥ}_{M}}\approx \overset{k=N}{\underset{k=1}{\prod }}e^{-i{\tilde{\beta }}_{j}{\tilde{P}}_{Z_{X_{k}}}} \end{array}$$ (47)

### 5.7 Combining the cost and mixer operators in QAOA

The quantum system progresses by successively applying the cost and mixer operators in an alternating manner. After executing *P* such layers, the resulting evolution of the state is expressed as:


$$|\Psi \left(\tilde{\beta },\tilde{\gamma }\right)\rangle =e^{-i\tilde{\gamma_{p}}{\hat{ℋ}}_{C}}e^{-i\tilde{\beta_{p}}{\hat{ℋ}}_{M}}...e^{-i\tilde{\gamma_{1}}{\hat{ℋ}}_{C}}e^{-i\tilde{\beta_{1}}{\hat{ℋ}}_{M}}|\Psi \left(0\right)\rangle$$ (48)

The parameters $\tilde{\beta }$ and $\tilde{\gamma }$ are tuned using classical optimization methods to reduce the expected value of the cost Hamiltonian, effectively steering the quantum system toward an optimal solution. These operations are repeated for *p* layers, with p controlling the number of alternating steps between the cost and mixer operators. The fully parametrized circuit can be expressed as:


$$|\Psi \rangle =\prod_{j=1}^{p}e^{-i\tilde{\beta_{j}}{\hat{ℋ}}_{M}}e^{-i\tilde{\gamma_{j}}{\hat{ℋ}}_{C}}\Psi \left(0\right)\rangle$$ (49)

The quantum state |Ψ〉 represents the outcome after applying *p* layers of operations. This quantum circuit, known as the ansatz, is defined by 2*p* tunable parameters, $\tilde{\gamma }$ and $\tilde{\beta }$. For each encoding, our goal is to minimize the expectation value of ${Ĥ}_{C}$, written as,


$$\begin{array}{l} \langle {Ĥ}_{C}\rangle =\langle \text{Ψ}\left(\tilde{\beta },\tilde{\gamma }\right)|{Ĥ}_{C}|\text{Ψ}\left(\tilde{\beta },\tilde{\gamma }\right)\rangle \end{array}$$ (50)

### 5.8 Classical parameter optimization

Once the quantum circuit is constructed, the parameters ${\tilde{\beta }}_{i}$ and ${\tilde{\gamma }}_{i}$ are adjusted using a classical optimization strategy to minimize the expected value of the objective function F. This expectation is usually non-convex, so we iteratively improve the parameters using classical heuristic algorithms (we opted for COBYLA). We obtain an approximate solution to the initial optimization problem by measuring the quantum circuit after the optimization process concludes.

### 5.9 Inverse reconstruction process

Following QAOA optimization, the Solver returns the optimal solutions as a set of selected undirected edges corresponding to the proposed SAR formulation (Figures 4A–C). A directed routing solution is recovered through a graph traversal procedure. First, each depot-connected edge initializes a vehicle route, and successive adjacent edges are traversed until the depot is reached again, thereby recovering one complete vehicle tour. This procedure is repeated for all depot-connected edge sets until every selected edge has been visited. The recovered route orientation is not unique because each undirected cycle can be traversed in either clockwise or counterclockwise direction. However, this ambiguity does not affect the solution quality. Since the underlying problem is a symmetric VRP((*c*_*ij*_ = *c*_*ji*_), traversing a tour in either direction yields an identical objective value. Consequently, every reconstructed directed route is a cost-equivalent representation of the same optimal undirected solution. Furthermore, the retained Subtour Elimination Constraints (SECs) guarantee that the selected edge set consists exclusively of depot-connected tours, thereby preventing disconnected subtours and ensuring the feasibility of the recovered routing problems. Consider Figure 4B, where the SAR-QAOA returns the 15-qubit bitstring 111111000000010. For instance with *n* = 6 nodes (including depot) and *k* = 3 vehicles, the corresponding set of undirected edges is $E=\left\{x_{01},x_{02},x_{03},x_{04},x_{05},x_{12},x_{13},x_{14},x_{15},x_{23},x_{24},x_{25},x_{34},x_{35},x_{45}\right\}$. According to the ordering of the extracted bitstring, the selected edge set is ${ℰ}^{*}=\left\{x_{01},x_{02},x_{03},x_{04},x_{05},x_{12},x_{35}\right\}$, which defines the partial feasible graph that is traversed by the SAR-based route extraction algorithm to obtain the individual vehicle routes.

**Figure 4:**
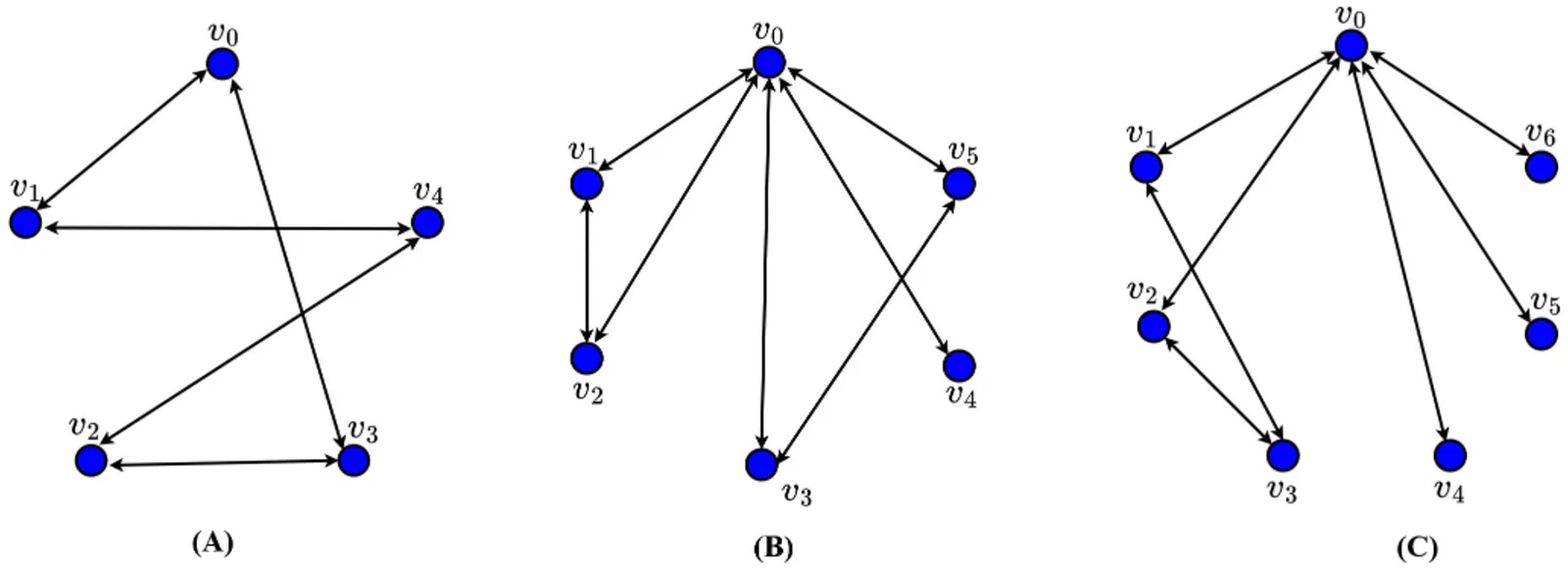
Visualization of optimal bitstings obtained from SAR-QAOA: **(A)** 5-node 1-vehicle minimum cost 123.43( 1010001110), **(B)** 6-node 3-vehicle minimum cost 133(111111000000010), **(C)** 7-node 4-vehicle minimum cost 95.7(110111010001000000000).

## 6 Time complexity

In our SAR-QAOA edge-based quantum routing optimization framework, the classical mapping procedure to translate the network routing into a QUBO problem requires a time complexity of $N+O\left(E^{2}\right)$, where N represents the number of nodes and *E* is the number of edges. The next step involves constructing and executing the QAOA Ansatz. In the QAOA circuit, initialization begins by applying Hadamard gates to each qubit, which takes O(E) time. The subsequent layers involve the application of parameterized *R*_*z*_ gates, which require O(1) time. Since *R*_*zz*_ gates commute when acting on distinct qubit pairs, they can be executed simultaneously. Under conditions of maximum parallel execution, this results in a time complexity of O(logE). Finally, the mixer Hamiltonian layer, composed of *R*_*x*_ gates, can also be applied in parallel, resulting in a time complexity of O(1). Therefore, the time complexity our SAR-QAOA Ansatz is $O\left(N^{2}\right)+O\left(E^{2}\right)+O\left(log\left(E\right)\right)=O\left(E^{2}\right)$. Moreover, $M_{it}$ is the number of consecutive iterations needed to optimize the parameters using a classical optimizer (in our case, COBYLA), and T is the number of distinct circuit executions it takes to sample the best solutions following parameter optimization. Finally, the overall time complexity for our SAR-QAOA approach is $\left(M_{it}+T\right)O\left(E^{2}\right)$.

## 7 Results and discussion

### 7.1 Computational study

Using Pennylane's Python framework, a number of simulations were run in order to confirm the efficacy of the suggested SAR-QAOA approach. An i9 processor with 64GB of RAM was used to run the experiment. Installing the required libraries, such as Pennylane (Xanadu, Toronto, Canada), NumPy (NumPy Developers, Austin, Texas, USA), and Matplotlib (Matplotlib Development Team, Austin, Texas, USA), and setting up a Python environment are prerequisites. Pennylane lightning.qubit is utilized in our study.

### 7.2 Performance metrics

To assess the performance of our proposed quantum approach, brute-force solutions are reported only as ground-truth references for small VRP instances to provide exact optimal values, while simulated annealing serves as the primary classical heuristic baseline for performance comparison. Furthermore, to highlight the advantages of the proposed SAR-QAOA framework, its performance is benchmarked against standard QAOA. The demonstrated results provide the exact optimum for small instances (see Figures 5a, b) and higher than 94% approximation solutions for all instances up to node (see Figure 5c). However, due to more qubit interactions for node 7, we achieved more than 70%, which is shown in Figure 5d that confirm that our method produces near-optimal solutions. To evaluate our approach's effectiveness quantitatively, we established the following key performance metrics. Compared to the classical Simulated Annealing, the cost variation $E_{SAR}^{b}\left(\%\right)$ and $E_{SAR}^{a}\left(\%\right)$ percentages (Equations 51 and 52) indicate that our hybrid quantum-classical approach has expanded.


$$\begin{array}{l} E_{SAR}^{b}\left(\%\right)=ESAR_{best}\left(\%\right)=|\frac{\left(SAR_{best}-OS\right)}{OS}|*100 \end{array}$$ (51)

$$\begin{array}{l} E_{SAR}^{a}\left(\%\right)=ESAR_{avg}\left(\%\right)=|\frac{\left(SAR_{avg}-OS\right)}{OS}|*100 \end{array}$$ (52)

where:

• *SAR*_*best*_ is the chosen best optimal solution obtained from the set of samples by the Hybrid quantum SAR-QAOA approach over *N* runs.
• *SAR*_*avg*_ is the average of all solutions obtained from the SAR-QAOA approach over *N* runs.
• *OS* is the Optimal Solution obtained by the classical simulated annealing method.

**Figure 5:**
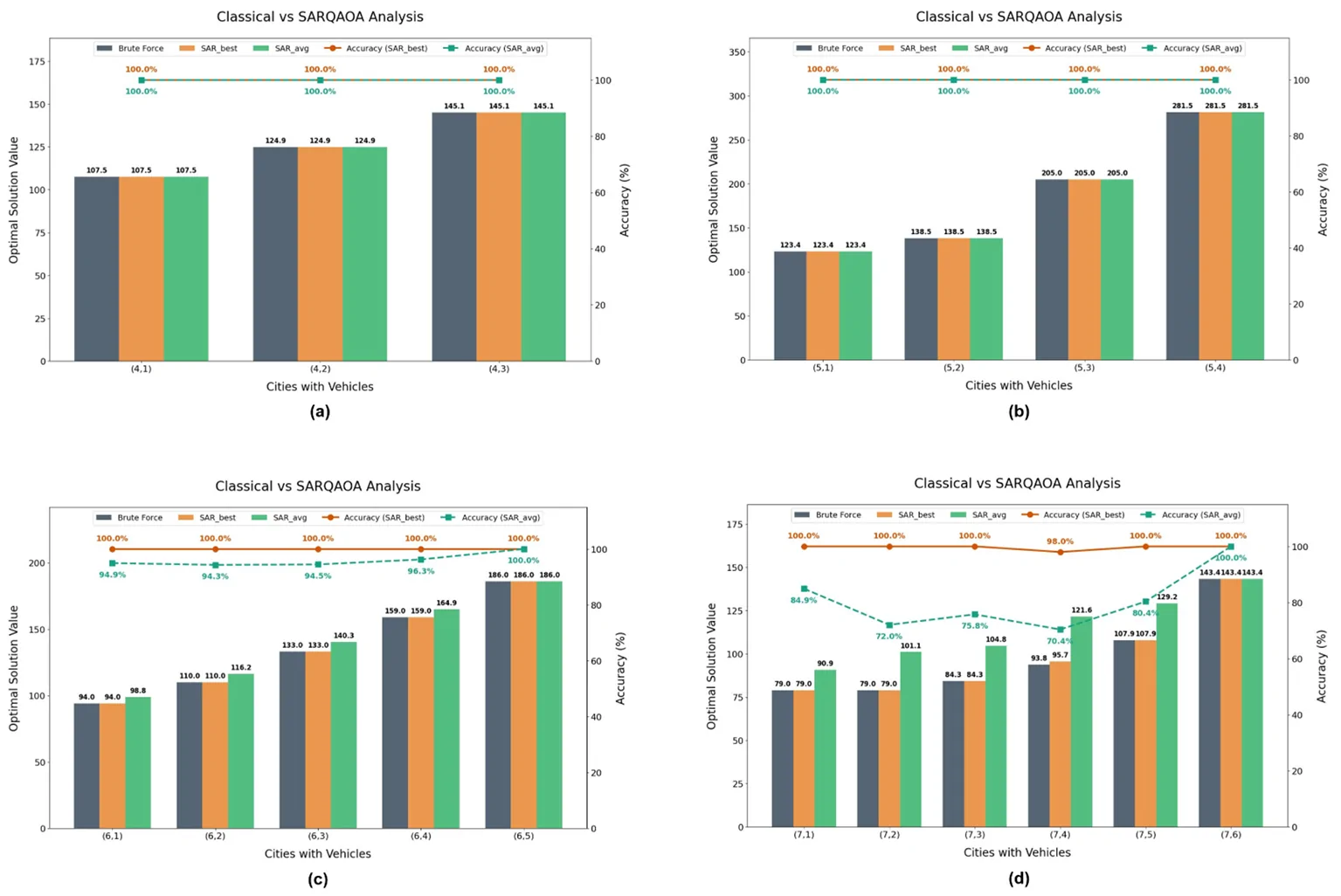
Performance and accuracy analysis of **(a)** *n* = 4 (6 qubits). **(b)** *n* = 5 (10 qubits). **(c)** *n* = 6 (15 qubits). **(d)** *n* = 7 (21 qubits).

The quantum-enhanced solution's proximity to the classical optimal solution is measured by the Approximation Ratio (AR) (Equations 53 and 54):


$$\begin{array}{l} {AR}_{b}=\frac{SAR_{best}}{OS},\;{AR}_{a}=\frac{SAR_{avg}}{OS} \end{array}$$ (53)

$$\begin{array}{l} SD\left(\sigma \right)=\sqrt{\frac{\sum_{i=1}^{N}\left(SAR_{i}-SAR_{avg}\right)}{N}} \end{array}$$ (54)

The Hybrid SAR-QAOA technique offers a nearly ideal solution when the approximation ratio is closer to 1.0, indicating that it produced competitive solutions.

### 7.3 Discussion

We tested our proposed SAR technique via a Hybrid QAOA approach on a vehicle routing instance from Azad et al. (2023) with *n* = 4, 5, 6, 7 nodes and *k* = 1, 2, ..., *n*−1 vehicles to examine the performance of our proposed SAR-QAOA method. The number of measurement shots(T) is varied as 5,000, 10,000, 50,000, 100,000, while the QAOA depth(P), denoted as P is incremented from 1 to 5. Each configuration is executed for a single experimental run to observe the impact of both shot count and circuit depth on solution convergence, computational stability, and energy optimization. The Optimal solution is the bitstring with the lowest cost found among all samples, regardless of its frequency. The lowest-cost solution is often optimal, even if it's rare.

In Figures 6, 7, we present the outcomes of distinct vehicle routing instances up to 21 qubits for the sake of conciseness. The optimal solutions are indicated by the low heat map hues, which also represent the lowest costs. The optimal beta and gamma parameter combinations are shown by a tiny rounded dark red circle. These combinations provided the bitstring (see Figures A1a–r in Appendix) that matched the most appropriate route, as the circuits output in each case of Figures 6, 7. Finding the optimum value ($\tilde{\gamma }$, $\tilde{\beta }$) is difficult since the heatmaps (Figure 7) show more shades, which is due to the fact that there are more pathways involved. Nevertheless, SAR-QAOA continues to approach the optimal bitstring solution.

**Figure 6:**
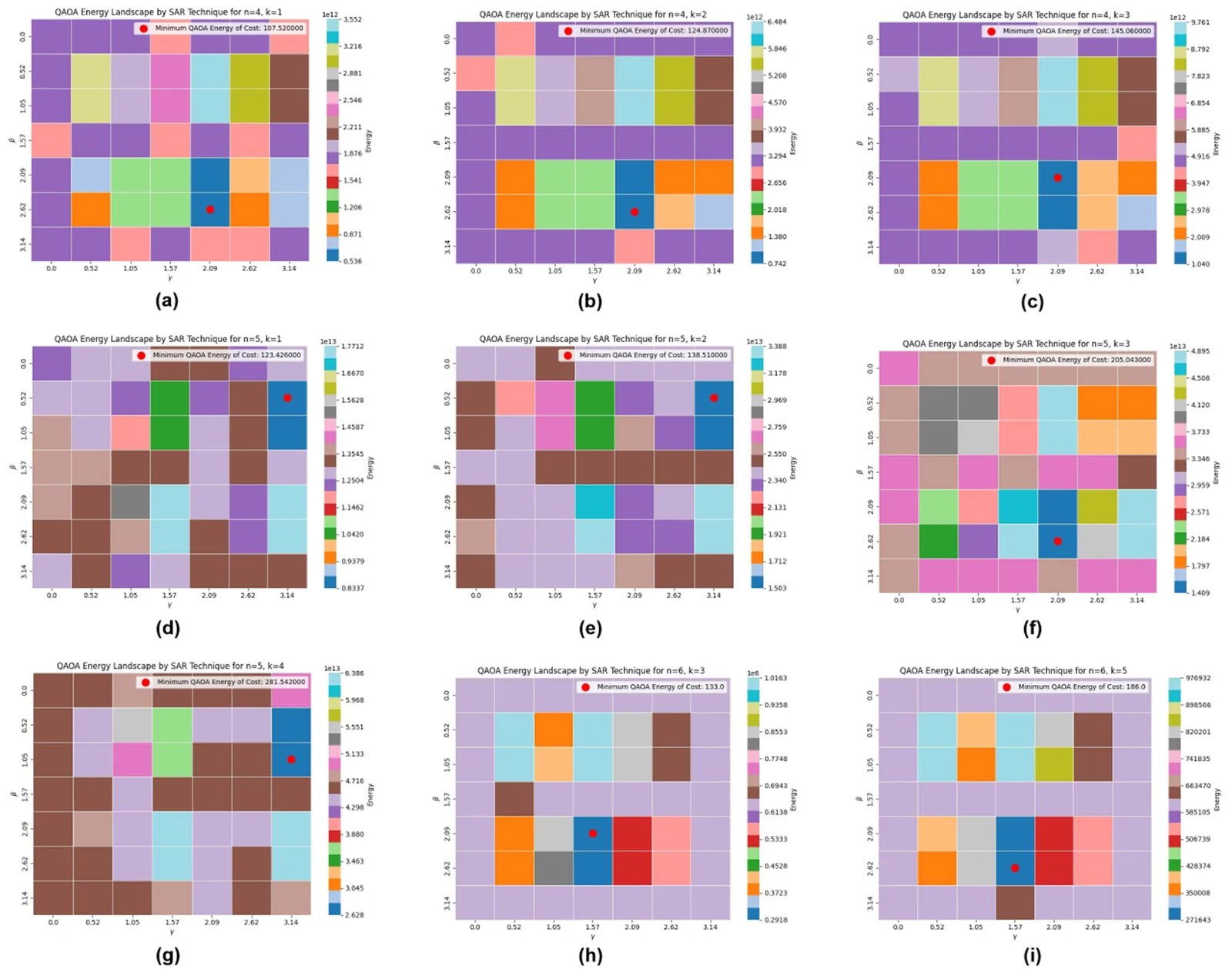
Various combinations of $\tilde{\gamma }$ and $\tilde{\beta }$ for different problem sizes (up to 15 qubits). **(a)** *n* = 4, *k* = 1, **(b)** *n* = 4, *k* = 2, **(c)** *n* = 4, *k* = 3, **(d)** *n* = 5, *k* = 1, **(e)** *n* = 5, *k* = 2, **(f)** *n* = 5, *k* = 3, **(g)** *n* = 6, *k* = 1, **(h)** *n* = 6, *k* = 3, **(i)** *n* = 6, *k* = 5.

**Figure 7:**
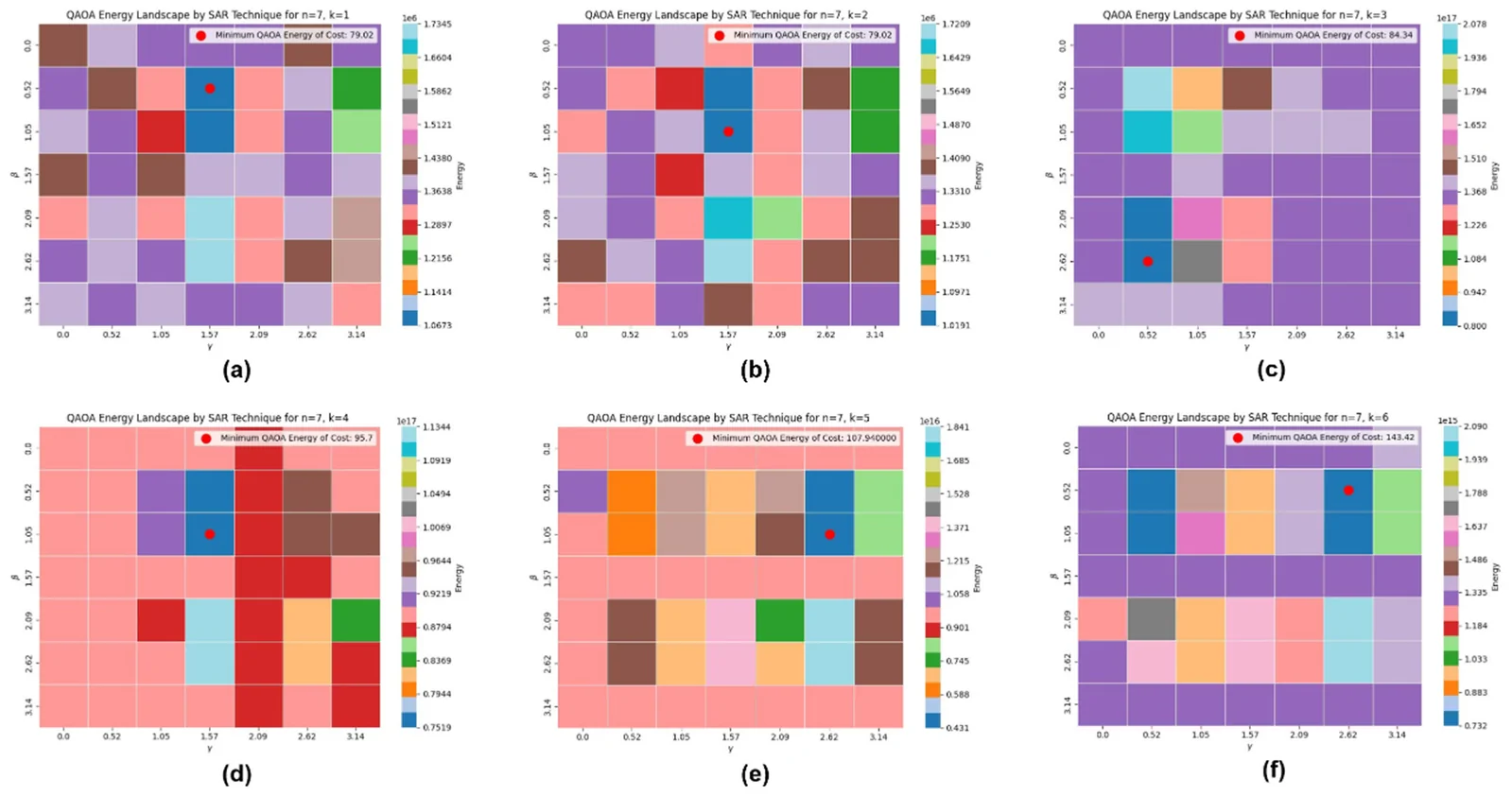
Various combinations of $\tilde{\gamma }$ and $\tilde{\beta }$ for *n* = 7 (21qubits). **(a)** *k* = 1, **(b)** *k* = 2, **(c)** *k* = 3, **(d)** *k* = 4, **(e)** *k* = 5, **(f)** *k* = 6.

The results obtained from running the Hybrid QAOA quantum circuit via our proposed SAR technique provide insights into the effectiveness and scalability of quantum computing for vehicle routing optimization tasks. The final sampling using the trained parameters resulted in a distribution of results, shown in Figures A1a–r in Appendix. The bitstring has a value of 1, and the remaining variables are 0. This outcome suggests that the algorithm was able to identify the optimal solution with a certain probability, not necessarily high, because of the stochastic nature of the algorithm. However, the probability distribution also shows that near-optimal solutions were sampled with non-negligible frequencies.

Table 4 presents the comparative results of the proposed SAR-QAOA across a series of Vehicle Routing Problem(VRP) instances. The experiments were performed for increasing network sizes (|V|) and varying numbers of vehicles (*k*). Each configuration was executed independently for *N* = 20 runs. The results were analyzed in terms of the best (*SAR*_*best*_, *QAOA*_*best*_) and average (*SAR*_*avg*_, *QAOA*_*avg*_) costs, their respective percentage deviations ($E_{SAR}^{b},E_{QAOA}^{b}$, $E_{SAR}^{a},E_{QAOA}^{a}$), approximation ratio (AR), and standard deviation (σ).

**Table 4:** Performance comparison of SAR-QAOA with classical and standard quantum baselines for CVRP instances.

Instance			Classical		Standard QAOA							COBYLA		Proposed SAR-QAOA							
|V|	k	Runs	Brute-force	SA (OS)	Q	QAOA_best_	QAOA_avg_	$E_{QAOA}^{b}$	$E_{QAOA}^{a}$	σ	AR	${\text{QAOA}}_{\text{best}}$	${\text{QAOA}}_{\text{avg}}$	Q	SAR_best_	SAR_avg_	$E_{SAR}^{b}$	$E_{SAR}^{a}$	σ	AR_*b*_	AR_*a*_
4	1	20	107.52	107.52	12	107.52	108.83	0.00	1.22	4.03	1.01	2.09	2.62	6	107.52	107.52	0.00	0.00	0.00	1.00	1.00
4	2	20	124.87	124.87	12	124.87	125.86	0.00	0.79	1.39	1.01	2.09	2.62	6	124.87	124.87	0.00	0.00	0.00	1.00	1.00
4	3	20	145.06	145.06	12	145.06	145.06	0.00	0.00	0.00	1.00	2.09	2.09	6	145.06	145.06	0.00	0.00	0.00	1.00	1.00
5	1	20	123.43	123.43	20	NFS	NFS	–	–	–	–	3.14	0.52	10	123.43	123.43	0.00	0.00	0.00	1.00	1.00
5	2	20	138.51	138.51	20	NFS	NFS	–	–	–	–	3.14	0.52	10	138.51	138.51	0.00	0.00	0.00	1.00	1.00
5	3	20	205.04	205.04	20	NFS	NFS	–	–	–	–	2.09	2.62	10	205.04	205.04	0.00	0.00	0.00	1.00	1.00
5	4	20	281.54	281.54	20	NFS	NFS	–	–	–	–	3.14	1.05	10	281.54	281.54	0.00	0.00	0.00	1.00	1.00
6	1	20	94.00	94.00	30^†^	NFS	NFS	–	–	–	–	1.57	2.09	15	94.00	98.75	0.00	5.05	6.83	1.00	1.05
6	2	20	110.00	110.00	30^†^	NFS	NFS	–	–	–	–	1.57	2.09	15	110.00	116.25	0.00	5.68	7.33	1.00	1.06
6	3	20	133.00	133.00	30^†^	NFS	NFS	–	–	–	–	1.57	2.09	15	133.00	140.35	0.00	5.53	5.88	1.00	1.06
6	4	20	159.00	159.00	30^†^	NFS	NFS	–	–	–	–	1.57	2.62	15	159.00	164.95	0.00	3.74	7.62	1.00	1.04
6	5	20	186.00	186.00	30^†^	NFS	NFS	–	–	–	–	1.57	2.62	15	186.00	186.00	0.00	0.00	0.00	1.00	1.00
7	1	20	79.02	79.02	42^†^	NFS	NFS	–	–	–	–	1.57	0.52	21	79.02	90.92	0.00	15.06	10.90	1.00	1.15
7	2	20	79.02	79.02	42^†^	NFS	NFS	–	–	–	–	1.57	1.05	21	79.02	101.11	0.00	27.95	15.79	1.00	1.28
7	3	20	84.34	84.34	42^†^	NFS	NFS	–	–	–	–	0.52	2.62	21	84.34	104.76	0.00	24.21	15.21	1.00	1.24
7	4	20	93.82	93.82	42^†^	NFS	NFS	–	–	–	–	1.57	1.05	21	95.7	121.61	2.00	29.62	14.13	1.02	1.30
7	5	20	107.94	107.94	42^†^	NFS	NFS	–	–	–	–	2.62	1.05	21	107.94	129.15	0.00	19.65	14.91	1.00	1.20
7	6	20	143.42	143.42	42^†^	NFS	NFS	–	–	–	–	2.62	0.52	21	143.42	143.42	0.00	–	–	1.00	–

|V|, Number of cities; k, Number of vehicles; Runs, Independent executions; OS, Optimal Solution; SA, Simulated Annealing; γ, β, Optimized parameters; Q, Number of Qubits; AR, Approximation Ratio; −, Undefined ; ^†^Practical statevector simulation is limited in standard QAOA; NFS, No Feasible Solution found.

#### 7.3.1 Performance of instances

For smaller routing configurations (|V|=4 and 5), SAR-QAOA precisely recreated the OS results, producing zero deviation $\left(E_{SAR}^{b}(\%)=E_{SAR}^{a}(\%\right)$=$E_{SAR}^{a}\left(\%\right)$=0%) and a perfect approximation ratio (*AR* = 1.0). Furthermore, the standard deviation was zero across all separate runs, indicating algorithmic determinism and stability in low-dimensional qubit domains (6–10 qubits).

For 6-node routing, SAR-QAOA utilizes 15 qubits and consistently converges to best solutions matching OS values without deviations. The approximation ratios of *SAR*_*best*_ and *SAR*_*avg*_ reveal more than 97% and 94%, respectively, uniformly across all instances, which guarantees the algorithmic stability of SAR-QAOA optimization. The standard deviation oscillated between 5.88 and 7.62, and $E_{SAR}^{a}\left(\%\right)$ was less than or equal to 5.7%, caused by the stochastic nature and random parameter initialization in the QAOA ansatz. The marginal path difference between quantum and classical solutions arises primarily from the stochastic nature of quantum measurements and the finite number of COBYLA optimization iterations employed.

Even though the average percentage error increases from 15.06% to 27.95% due to the complexity of the solution space, the influence of parameters, and quantum sampling noise, our *SAR*_*best*_ keeps the best solution since the number of qubits increases to 21 for the scaled routing size of 7 nodes up to 3 vehicles. A tiny variation in $E_{SAR}^{b}\left(\%\right)$=2% and SD(σ)=14.13 implies that random parameter updates are necessary to stabilize convergence when the vehicle number increases to 4. The algorithm reached a non-feasible solution (NFS) for the vehicle count increased to 6 in a 7-node instance, likely due to the higher qubit interactions required during classical simulation.

#### 7.3.2 Sampling analysis

In Table 5, the number of measurements (T), QAOA Layer depth (P), and the maximum iteration of COBYLA optimizer $\left(M_{it}\right)$ are recorded. The bitstrings that are reported correspond to the solutions obtained from sampled outcomes of possible low cost, rather than those with the highest measurement frequency. Our approach consistently proves the convergence by few shots 5,000 to 10,000 with shallow circuit depth(P) 1 to 3 with optimizer convergence within 32 iterations, resulting in bitstring 101101 in Figure A1a in Appendix and 111000 in Figure A1c in Appendix that matched with best routes of OS values in Table 4. Moreover, deeper circuits and higher sampling counts, such as 10, 000 to 50, 000 shots, are needed to yield the optimal solution of routing size 6 with up to 5 vehicles to support stability. Figure A1j in Appendix the bitstring 11111000000010 shows the robustness of the SAR encoding in preserving problem symmetry under increased dimensionality.

**Table 5:** Sampling results obtained using the SAR-QAOA approach.

Figure	Instance	T	Probability	P	$M_{it}$	Optimal bitstring
Figure A1a in Appendix	(4,1)	5,000	0.77	1	32	101101
Figure A1b in Appendix	(4,2)	10,000	0.42	1	32	111001
Figure A1c in Appendix	(4,3)	5,000	1.00	1	32	111000
Figure A1d in Appendix	(5,1)	5,000	0.24	3	32	1010001110
Figure A1e in Appendix	(5,2)	50,000	0.16	1	32	1011000110
Figure A1f in Appendix	(5,3)	5,000	0.74	1	32	1111000100
Figure A1g in Appendix	(5,4)	5,000	1.00	1	32	1111000000
Figure A1h in Appendix	(6,1)	10,000	0.034	2	32	100101000001110
Figure A1i in Appendix	(6,2)	20,000	0.077	2	32	100111000100010
Figure A1j in Appendix	(6,3)	50,000	0.067	1	32	111111000000010
Figure A1k in Appendix	(6,4)	10,000	0.417	3	32	111110000000010
Figure A1l in Appendix	(6,5)	5,000	1.00	1	32	111110000000000
Figure A1m in Appendix	(7,1)	50,000	0.067	3	32	000011110000100010010
Figure A1n in Appendix	(7,2)	20,000	0.167	2	32	000111110000100010000
Figure A1o in Appendix	(7,3)	100,000	0.111	2	32	010111110000000010000
Figure A1p in Appendix	(7,4)	10,000	0.250	1	300	110111010001000000000
Figure A1q in Appendix	(7,5)	30,000	0.500	1	300	111111010000000000000
Figure A1r in Appendix	(7,6)	10,000	1.00	1	64	111111000000000000000

When investigating larger problem instances with seven customers (21 qubits), the optimization landscape became substantially more complex and showed greater susceptibility to quantum noise. The classical optimizer iterated between 64 and 300, and shots ranged from 10,000 to 100,000 per execution to achieve convergent solutions. This aligns the behavior with theoretical predictions for high-dimensional cost surfaces, wherein discovered bit patterns show increasing sparsity while preserving discernible structural regularity. Although the most computationally intensive case of 7 customers (21 qubits) imposed substantial quantum resource limitations, SAR-QAOA still maintained its ability to generate practical, cost-effective routing solutions. Taken together, these findings establish that SAR-QAOA effectively improves the resource efficiency of the QUBO formulation while maintaining competitive solution quality for symmetric vehicle routing problems. The presented results validate the feasibility of the proposed approach and establish a stepping stone for further investigations on larger problem instances and Near-term quantum hardware.

#### 7.3.3 Scalability and quantum feasibility

Current quantum computing platforms are constrained by limited qubit availability, coherence times, and gate fidelities, which restrict the size of optimization problems that can be executed efficiently. Moreover, the primary objective of this study is to validate the proposed SAR-QAOA formulation and demonstrate its improved resource efficiency through reduced qubit requirements. As shown in the experimental results, the proposed SAR technique substantially reduces qubit overhead compared with conventional slack-based formulations, yielding a more compact QUBO representation better suited to quantum optimization.

Although problem instances involving seven customers are computationally tractable for classical optimization methods, they provide a meaningful benchmark for evaluating the resource requirements of gate-based quantum simulation. The benefit of the proposed SAR-QAOA formulation can be illustrated through the memory requirements of state-vector simulation. A conventional full-graph formulation for *N* = 7 requires *N*^2^ = 49 qubits. Simulating a 49-qubit state vector requires storing 2^49^ complex amplitudes, or about 8 PB of memory, which exceeds the capabilities of most conventional high-performance computing systems. Conversely, the proposed SAR-QAOA formulation reduces the qubit requirement to 21, for which the corresponding state vector requires only approximately 32 MB of memory. This substantial reduction in memory requirements enables efficient simulation and verification of the proposed formulation on classical quantum simulators and highlights its potential for future implementation on more capable quantum hardware as the technology continues to mature.

### 7.4 Limitations and future work

The proposed SAR approach suits symmetric cost structures, as typically found in road vehicle routing. Extending the method to asymmetric cases, such as air routing and marine routing where directional costs differ, is identified as a potential avenue for future enhancement. While the Pennylane quantum simulator can handle simulations with limited qubits representing edges in our model, this study was restricted to four test cases comprising graphs with 4 to 7 nodes. This restriction was primarily due to the higher computational complexity and resource consumption of simulating larger QAOA circuits using the SAR method. At this stage, we could not identify any viable solutions, so we discontinued further analysis. The objective of the computational study is to validate the correctness, optimality-preserving characteristics, and qubit reduction achieved by the proposed SAR formulation rather than to demonstrate large-scale computational performance. Future research will investigate the proposed formulation on larger benchmark instances using quantum high-performance simulators and hardware or hybrid quantum-classical computing platforms, together with comparisons against state-of-the-art exact and heuristic VRP solvers.

## 8 Conclusion

In this work, we demonstrated the application of a hybrid QAOA algorithm for solving the Vehicle Routing Problem by introducing a Symmetric Arc Reduction (SAR) formulation. The proposed technique was designed to minimize total travel cost while reducing the qubit requirements of the corresponding QUBO formulation, providing a more resource-efficient formulation for quantum optimization. Our experimental evaluation, which benchmarked SAR-QAOA against both Standard QAOA and classical Simulated Annealing (SA), yields two critical insights regarding resource scaling and solution feasibility. Although classical heuristics currently retain an advantage in execution speed, SAR-QAOA consistently matched the Optimal Solution (OS) found by classical Simulated Annealing and the brute-force method for instances up to 5 nodes and produced high-quality near-optimal solutions for the larger tested instances(nodes 6,7).

In summary, quantum computing is only in its infancy because of restrictions not only on the number of qubits, but also on their control errors. While we use the SAR technique with QAOA on VRP in this research, it is important to note that our methodology can be applied to a wider spectrum of problems, for example, the two-echelon vehicle routing problem and network topology. Extending our proposed technique to heterogeneous vehicle routing problems is a key direction for future research. Quantum computing has the promising computational power to solve the vehicle routing problem on a suitably large scale, as it has an increasing number of qubits and developing error correction methods.

## Figures and Tables

**Figure A1:**
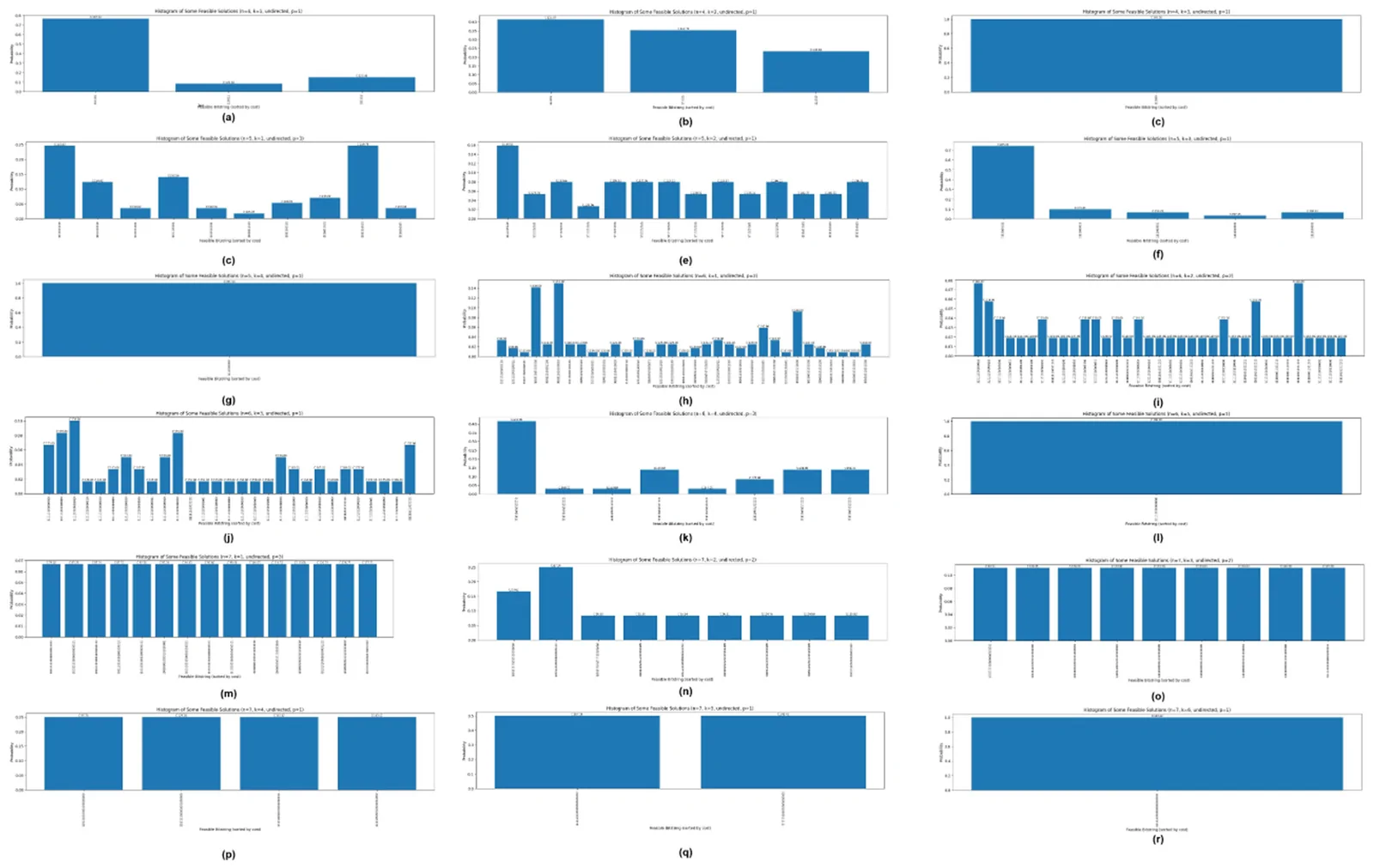
Probability distributions obtained from QAOA simulations for various problem sizes and depths. **(a)** *n* = 4, *k* = 1 (6 qubits) through **(r)** *n* = 7, *k* = 6 (21 qubits).

## Data Availability

The original contributions presented in the study are included in the article/supplementary material, further inquiries can be directed to the corresponding author.
